# Dynamic Interpretation of Hedgehog Signaling in the *Drosophila* Wing Disc

**DOI:** 10.1371/journal.pbio.1000202

**Published:** 2009-09-29

**Authors:** Marcos Nahmad, Angelike Stathopoulos

**Affiliations:** 1 Department of Control and Dynamical Systems, California Institute of Technology, Pasadena, California, United States of America; 2 Division of Biology, California Institute of Technology, Pasadena, California, United States of America; Stanford University, United States of America

## Abstract

Morphogens are classically defined as molecules that control patterning by acting at a distance to regulate gene expression in a concentration-dependent manner. In the *Drosophila* wing imaginal disc, secreted Hedgehog (Hh) forms an extracellular gradient that organizes patterning along the anterior–posterior axis and specifies at least three different domains of gene expression. Although the prevailing view is that Hh functions in the *Drosophila* wing disc as a classical morphogen, a direct correspondence between the borders of these patterns and Hh concentration thresholds has not been demonstrated. Here, we provide evidence that the interpretation of Hh signaling depends on the history of exposure to Hh and propose that a single concentration threshold is sufficient to support multiple outputs. Using mathematical modeling, we predict that at steady state, only two domains can be defined in response to Hh, suggesting that the boundaries of two or more gene expression patterns cannot be specified by a static Hh gradient. Computer simulations suggest that a spatial “overshoot” of the Hh gradient occurs, i.e., a transient state in which the Hh profile is expanded compared to the Hh steady-state gradient. Through a temporal examination of Hh target gene expression, we observe that the patterns initially expand anteriorly and then refine, providing in vivo evidence for the overshoot. The Hh gene network architecture suggests this overshoot results from the Hh-dependent up-regulation of the receptor, Patched (Ptc). In fact, when the network structure was altered such that the *ptc* gene is no longer up-regulated in response to Hh-signaling activation, we found that the patterns of gene expression, which have distinct borders in wild-type discs, now overlap. Our results support a model in which Hh gradient dynamics, resulting from Ptc up-regulation, play an instructional role in the establishment of patterns of gene expression.

## Abstract 2

*Drosophila* cell response to the Hedgehog morphogen depends not just on a precise measurement of morphogen concentration at any given time, but instead on the history of cell exposure to morphogen.

## Author Summary

Cells in a developing embryo require information about their position with respect to other cells in order to function and differentiate appropriately. The predominant current model suggests that cells acquire this positional information by measuring the local concentration of signaling molecules called morphogens. In the developing wing of the fruit fly *Drosophila melanogaster*, the signaling molecule Hedgehog is distributed in a concentration gradient along the anterior–posterior axis and specifies at least three different domains of gene expression. Although there is a clear correlation between the Hedgehog extracellular gradient and the location of these gene expression domains, a direct correspondence between the borders of these patterns and local Hedgehog concentrations has not been demonstrated. Using computer simulations of the Hedgehog signaling network, we provide evidence that direct sensing of a static morphogen gradient is not sufficient to specify the observed cellular responses. We propose instead an alternative model for how cells define their position by interpreting the Hedgehog gradient. Our model suggests that, rather than simply make a precise measurement of the morphogen concentration to which cells are exposed at any given time, cells instead take into account their previous history of Hedgehog exposure. We provide experimental evidence that supports our model, and conclude that gradient dynamics are required for Hedgehog-dependent patterning in this system.

## Introduction

**Figure 1:**
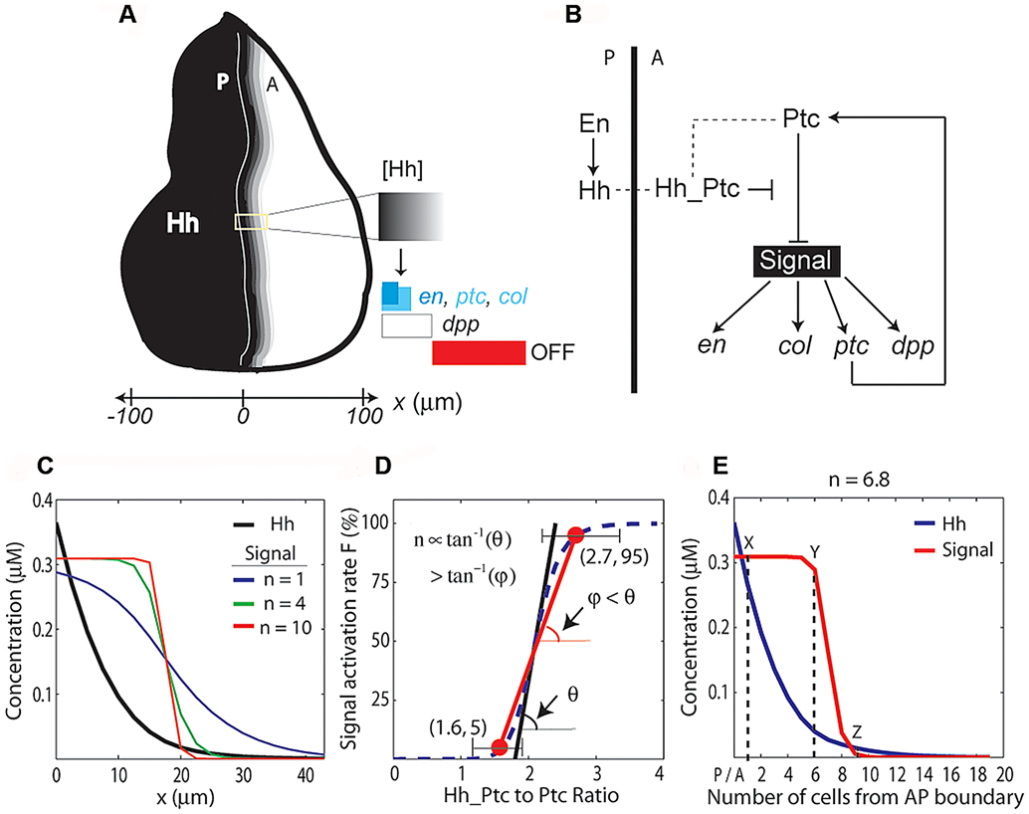
Mathematical modeling proposes that the Hh steady-state gradient is translated into a step-like signal response. (A) In the wing disc, Hh emanates from the posterior (P) compartment and forms a concentration gradient within the anterior (A) compartment to organize three different domains of gene expression: (I) *engrailed* (*en*), *patched* (*ptc*) and *collier* (*col*): blue; *en* is initially expressed in a narrow domain (dark blue), but it later expands to encompass the same domain as *ptc* and *col* (light blue). (II) *decapentaplegic* (*dpp*): white. (III) Cells beyond the *dpp* domain do not respond to the signal (OFF: red). Here and in subsequent figures, wing discs are oriented with posterior to the left and position along the AP axis is measured relative to the AP boundary (*x* = 0). (B) Simplified gene network of Hh signaling in the *Drosophila* wing disc as modeled in this study. Arrows represent activation; blunt-end lines represent repression. (C) Simulated steady-state (ss) profiles of [Hh] and [Signal]. The [Hh]_ss_ profile (black curve) is approximately invariant to changes in the parameter n, whereas the [Signal]_ss_ profile is qualitatively different for different values of n. (D) Estimation of n from published data [17]. The dashed line is the graph of 
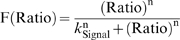
 with 
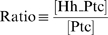
. Ratio = 1.6 produces no signaling response (∼5%), whereas Ratio = 2.7 gives full activation of the pathway (∼95%) [17]. A lower-bound value of n can be estimated by the slope of the line through these data points [red line; 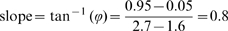]. Therefore, 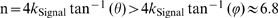. The minimum lower-bound estimate, taking into account error bars (drawn according to published data [17]), is n>4.0. (E) Simulated steady-state profiles of [Hh], blue line, and [Signal], red line, for n = 6.8. Cell position relative to the AP boundary is indicated on the horizontal axis (*x*-axis). “X”, “Y”, and “Z” denote three arbitrary positions along the *x*-axis, but were chosen to illustrate that [Signal] has a step-like profile. The differences in [Hh] at these positions depend on the chosen parameters (Table S1) and are presented here only as an example (see Text S1).

Pattern formation in many developing systems depends on the formation and interpretation of morphogen gradients [1]. The classical model of pattern formation conveyed by morphogens is typically illustrated by Wolpert's French Flag model [2]. In the context of Wolpert's model, cells capable of sensing an extracellular gradient will adopt one of three different regulatory states depending on their local readout of the morphogen concentration. Based only on the input (ligand gradient) to output (target gene expression) relationship, several signaling molecules appear to operate in the context of Wolpert's Classical Morphogen model [3]–[5]; however, the underlying mechanisms regarding how gradients of these signaling molecules are translated into discrete patterns of gene expression remain unclear. One example of such a signaling molecule is Hedgehog (Hh), as in the *Drosophila* wing disc, the Hh distribution clearly correlates with gene expression patterns (Figure 1A) [6]. Yet, it has not been demonstrated definitively that different Hh concentrations define the positions of distinct borders of gene expression patterns.

Hedgehog molecules are secreted proteins that control patterning pervasively during animal development [7]. In the *Drosophila* wing imaginal disc, *hh* is expressed exclusively in cells of the posterior compartment. After several posttranslational modifications, Hh is secreted from the posterior compartment, forming a concentration gradient within the anterior compartment with highest levels present at the anterior–posterior (AP) boundary. Although the range of Hh signaling is short compared to that of Decapentaplegic (Dpp), 10–15 cells compared with approximately 40 cells, respectively, at least three different patterns are established by Hh signaling [6],[8]–[11]. Target genes activated by Hh signaling include *dpp*, *collier* (*col*), *patched* (*ptc*), and *engrailed* (*en*) (Figure 1A).

A simplified network of genetic interactions involved in the Hh signaling pathway is shown in Figure 1B. Hh signaling is maintained in a default OFF state by the Hh receptor Patched (Ptc), which is constitutively expressed in cells of the A compartment. Ptc expression inhibits the activation of the transmembrane protein Smoothened (Smo) through a mechanism that is still not well understood and is likely indirect [12],[13]. Extracellular Hh binds to Ptc and induces its internalization and degradation. In the absence of Ptc, Smo accumulates and is phosphorylated, a step required to activate Hh signaling [14],[15]. The details regarding how phosphorylated Smo (pSmo) results in activation of the pathway are complex and not well defined, but require Smo-mediated recruitment of a series of kinases that prevent processing of the transcription factor cubitus interruptus (Ci). In the absence of Hh or pSmo, Ci is cleaved, and one of the fragments, known as Ci75, acts to repress particular Hh target genes [16]. Activation of the Hh pathway stabilizes pSmo expression, which in turn inhibits the cleavage of Ci into Ci75, to permit full-length Ci to enter the nucleus and activate the transcription of Hh target genes.

Activation of Hh signaling in the wing disc appears to depend, not only on the concentration of free Ptc, but also on the amount of Ptc bound to Hh [17]. Thus, it has been proposed that transduction of Hh signaling depends on the ratio of liganded to unliganded Ptc [17]. Furthermore, an evolutionarily conserved property of the Hh signaling pathway is that *ptc*, the gene that encodes the Hh receptor, is transcriptionally up-regulated by Hh signaling (see Figure 1B). This feedback gives Ptc the dual function of both receiving the signal as well as limiting the spatial range of the Hh gradient [18].

In this study, we investigated the mechanisms by which the Hh gradient is interpreted to pattern the *Drosophila* wing disc. Our approach was to use mathematical modeling to formulate hypotheses that can be tested directly through experimentation. Surprisingly, our mathematical analysis suggested that the steady-state Hh gradient is insufficient to determine more than two gene expression patterns in a concentration-dependent manner. We propose that Hh-dependent Ptc up-regulation causes a transient expansion (or “overshoot”) of the Hh gradient before approaching its final distribution. Through experiments conducted in vivo, we provide evidence that this transient overshoot exists and that it is required to distinguish different spatial domains of gene expression in response to Hh. Taken together, our data suggest a new model of pattern formation, which takes into consideration gradient dynamics to explain Hh-dependent patterning of the wing disc.

## Results

### Mathematical Modeling of Hh Signaling Interpretation

We devised a mathematical model of Hh signaling based on the simplified network presented in Figure 1B (see Text S1 for further details). The dynamics of gene (and protein) concentrations along the AP axis are modeled using the following system of reaction-diffusion equations:
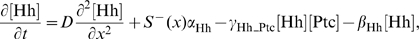
(1)

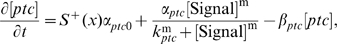
(2)

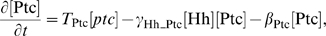
(3)

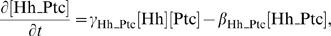
(4)

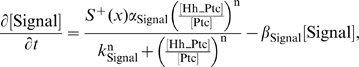
(5)where [Hh], [*ptc*], [Ptc], and [Hh_Ptc] are the concentrations of Hh, *ptc* (mRNA), Ptc (protein), and the Hh-Ptc complex, respectively. The coefficients α, β, γ, and T represent the rates of synthesis, degradation, complex formation, and translation, respectively. We use a system of coordinates centered on the AP boundary with the anterior compartment on the positive side (Figure 1A). S^+^(*x*) (or S^−^(*x*)) is a step function of the form *S^+^*(*x*) = 1 if *x*>0 [or *S^−^*(*x*) = 1 if *x*<0] and zero otherwise (see Text S1 for further model details).

We use the variable [Signal] (instead of a particular effector such as pSmo, for example) to represent the concentration of Hh signaling activity and assume that [Signal] reflects the propensity to activate Hh target gene expression. Our model of signal activation is based on the phenomenological observations that Hh-dependent gene expression depends on the ratio of liganded (Hh_Ptc) to unliganded Ptc (Equation 5) [17]. Unlike other models of Hh signaling conducted in the past [19]–[23], our model is not based on any particular molecular mechanism regarding how Ptc and Smo interact. A mechanistic model would require additional knowledge of the specific biochemical interactions, including how Ptc interacts with Smo and how Smo interacts with other pathway components, which are currently not completely understood. Our goal was to use mathematical modeling as a tool to formulate experimentally testable hypotheses, in order to study how a gradient of Hh is interpreted by a field of cells.

### Theoretical Analysis Suggests That the Steady-State Hh Gradient Is Interpreted as a “Switch-Like” Response

At steady state, the system of equations (Equations 1–5) reduces to a single second-order equation for [Hh] with boundary conditions (Equation S1 in Text S1), and all other concentrations can be written as a function of [Hh]. For example, setting the left-hand side of Equations 4 and 5 to zero, and substituting Equation 4 into 5, we obtain the following expression valid at steady state in the anterior compartment:
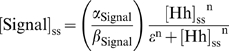
(6)with
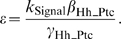



Equation 6 is a nonlinear input–output relationship between the (unbound) [Hh]_ss_ gradient and the [Signal]_ss_ profile, in which the subscript ss, refers to steady-state concentrations. It reveals that the steady-state interpretation of the Hh gradient, in terms of the [Signal] profile, depends qualitatively on the Hill coefficient n. For n∼1, an extracellular [Hh] gradient corresponds to a monotonic [Signal] gradient, but as n increases, the spatial distribution of [Signal] rapidly acquires a step-like profile (Figure 1C). Let F be the rate of Signal activation (first term on the right in Equation 5):
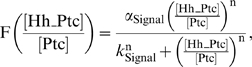
which is assumed to be a sigmoidal function of the [Hh_Ptc] to [Ptc] ratio [17], then the value of n is proportional to the slope of the line tangent to the graph of F at the point of half-maximal activation (Figure 1D). Thus, it is possible to obtain a lower-bound estimate of n using any two data points on this curve (see Text S1). Using published data [17], we estimated that n>6.8 (Figure 1D). The quantitative details of this approximation depend on several unknown parameters as well as the quantitative accuracy of the data used (see Text S1). Nevertheless, the qualitative behavior when n≫1 is clear. Most cells interpret either maximal signaling levels (ON state) or little to no signal (OFF state), and the transition between these two states is sharp (Figure 1E and Text S1). However, this analysis fails to explain how three (or more) domains of gene expression might be specified by the Hh gradient (see Figure 1A).

### Computer Simulations Suggest That Hh Gradient Dynamics May Contribute to Differential Gene Expression

Our mathematical analysis supports the hypothesis that at steady state, a graded extracellular input of [Hh] is interpreted into a switch-like profile of signal activation [Signal], in which two domains of gene expression could easily be supported by the ON versus OFF states. Although morphogens are often considered as static gradients or their transient dynamics are largely ignored [5],[24], recent studies have highlighted the importance of morphogen gradient dynamics in supporting differential gene expression [19],[25]–[27]. In order to reconcile the apparent switch-like profile of Hh signaling with the fact that at least three domains of gene expression are specified, we asked whether dynamics of the Hh gradient contribute to patterning of the *Drosophila* wing disc.

**Figure 2:**
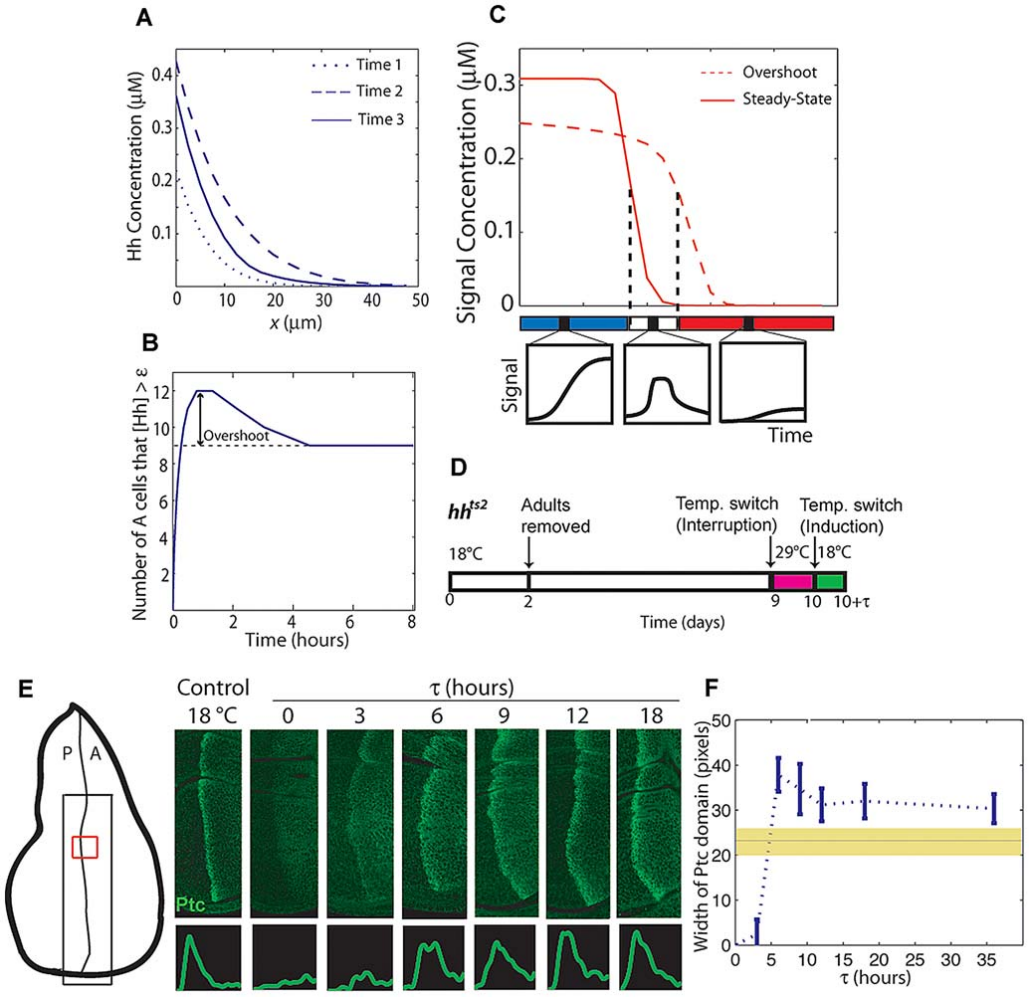
Temporal analysis of Hh signaling in vivo reveals the existence of a spatial overshoot. (A) Simulated profile of [Hh] gradient at three different time points: Time 1<Time 2<Time 3. The gradient transiently expands anteriorly (Time 2) and then refines towards its steady-state shape (Time 3). (B) Number of cells that experience higher concentrations of [Hh] than the ON/OFF switch threshold, ε, as a function of time based on the simulated [Hh] profiles. This diagram defines an overshoot, a spatial domain of cells that are exposed transiently to [Hh]. (C) The dashed and solid red lines represent the [Signal] profiles resulting from the “overshoot” and “steady-state” gradients. Cells experience three qualitatively different dynamic trajectories of [Signal] exposure depending on their spatial location (lower boxes). (D) Experimental setup to reinitialize the Hh gradient using a temperature sensitive *hedghog* allele (*hh^ts2^*; see Materials and Methods for details). Induction at 18°C to induce Hh signaling was conducted for different intervals of time (τ). (E) Ptc immunostaining of *hh^ts2^* homozygous discs from the experiment described in (D) for different induction times τ compared to a disc from a larva raised solely at 18°C. The large box in the illustration shows the entire region imaged; the small red box corresponds to a sample region used to generate the profiles using Protocol S1, displayed in the bottom panels. Discs were fixed, immunostained, and imaged under identical conditions (see Materials and Methods). (F) Width of the Ptc domains as the Hh gradient is developed. At least four discs at each time point were used. Mean widths were computed, and error bars indicate standard deviations (see Protocol S1). Yellow area indicates the width range for Ptc in control discs at 18°C.

We examined the dynamic establishment of the Hh gradient through numerical simulations of Equations 1–5 (see Materials and Methods). We observed that the gradient expands transiently to a position further from its steady-state distribution and then refines towards its source, the posterior compartment, as it approaches its final form (Figure 2A). This dynamic behavior of the gradient is most likely due to the dynamics of Hh-dependent Ptc expression. During the formation of the gradient, Ptc is expressed only at low levels throughout the anterior compartment, and therefore, the range of Hh is largely unrestricted by Ptc; once Ptc is up-regulated, then Hh mobility is affected. As a consequence, we observe that a transient expansion of the gradient, or overshoot, results. Cells in more anterior positions receive the Hh-dependent signal for a limited amount of time, but they no longer receive the signal once the gradient has reached its steady-state position (Figure 2B).

On the basis of this theoretical analysis, we proposed an Overshoot model to explain the three states observed in the system: (State 1) cells that are never exposed to Hh above a “switching threshold,” the level necessary to activate Hh signaling, are always in the OFF state (Figure 2C, red); (State 2) those cells that are only transiently exposed to levels above the switching threshold may transiently turn gene expression ON, but only certain genes subject to additional regulation will be able to maintain expression (Figure 2C, white); and (State 3) those cells that are exposed constantly to levels above the switching threshold exhibit an ON state (Figure 2C, blue). Interpretation of Hh signaling in the context of the Overshoot model requires only a single concentration threshold (the switching threshold) and takes into account each cell's dynamic history of Hh exposure (see Discussion).

Taken together, our mathematical modeling enabled us to formulate the following hypotheses. First, the Hh gradient at steady state is insufficient to generate more than two states, and, thus, Hh patterning cannot be explained using the Classical Morphogen model. Second, the formation of the gradient exhibits a transient overshoot. This dynamic behavior would be a consequence of the gene network architecture, in particular, it results from Hh-dependent up-regulation of Ptc. And third, that the overshoot is necessary to specify more than two domains of gene expression in the system. These hypotheses are experimentally testable, and once they are supported by in vivo experimentation, we contend that the details of the mathematical model (parameter values, equation terms, etc.) are less important.

### In Vivo Evidence for a Transient Expansion of the Hh Signaling Response

First, we designed an in vivo approach to test the existence and function of a Hh overshoot in wing disc patterning. Experimental evidence for the Hh gradient overshoot can be inferred from a recent study in which Hh was visualized using inducible Hh-GFP, though the existence of the overshoot was not highlighted by the authors [28]. We investigated whether or not an overshoot at the level of extracellular Hh protein would also have an effect on target gene expression.

To test this idea, we used a system in which Hh signaling in the wing disc can be reinitialized and target gene expression assayed in time through the use of a temperature-sensitive *hedgehog* allele, *hh^ts2^* [29] (Figure 2D). After 24 h at restrictive temperature, no Hh protein is detected by Western blot analysis, suggesting that Hh protein synthesis is impaired at the restrictive temperature (S. Eaton, personal communication). As the *ptc* gene itself is a target of Hh signaling, we investigated Ptc recovery in time after the Hh gradient is re-established (Figure 2D). We found that Hh-dependent Ptc expression transiently expands, followed by a posterior refinement (Figure 2E). The refinement observed is not simply due to changing the temperature, because no differences in Ptc expression are observed in equivalently treated wild-type discs (Figure S1). This overshoot of Ptc expression is observed as soon as 6 h after reinitialization of the gradient, and even after 36 h, refinement of the Ptc pattern is not complete (Figure 2F). The dynamic states of this pattern support that a Hh gradient overshoot exists and, more importantly, demonstrate that an overshoot can be detected at the level of target gene expression.

### Hh-Dependent Ptc Up-Regulation Is Required for the Establishment of Different Domains of Gene Expression

Previous studies have conclusively demonstrated that Ptc restricts the range of the Hh gradient [18]. As the Hh gradient expands further in *ptc* clones, we hypothesized that a Hh overshoot is likely to depend on signal-induced Ptc up-regulation. Therefore, we investigated how Hh patterning is affected in discs that lack the ability to up-regulate Ptc. *ptc* mutant animals die during embryogenesis but can be rescued, remarkably, by introducing ubiquitous levels of *ptc* through a Tubulin1α>*ptc*>Tubulin1α (TPT) transgene [18]. In *ptc* mutant discs carrying a copy of the TPT transgene (*ptc*−TPT; see Materials and Methods), it has been documented previously that Hh target genes are expressed in a broader domain compared to wild type [18], but no information regarding the relative positions of target genes has been reported.

**Figure 3:**
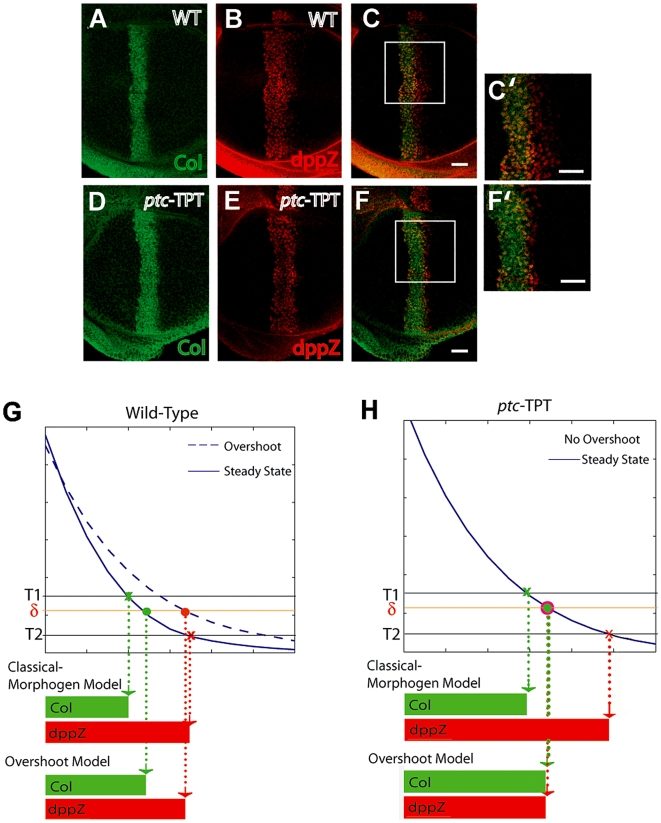
Hh-dependent Ptc is required for the specification of more than two domains of gene expression. (A–F) Immunostaining of third instar wing discs carrying the dppZ reporter using anti-Col ([A and D] green) and anti-β-gal ([B and E] red) antibodies. (C and F) Merge of images in (A and B) and (D and E), respectively. Stainings within wild-type (WT) discs or *ptc*− discs carrying a single copy of the TPT transgene are depicted in (A–C) and (D–F), respectively. (C′ and F′) 2.25× magnification of white boxed regions in (C and F), respectively. Scale bar indicates 10 µm. (G) Interpretation of the wild-type Hh gradient according to the Classical Morphogen model or Overshoot model; Col and dppZ are shown as examples. The Classical Morphogen model assumes the existence of concentration thresholds (T1 and T2) in the steady-state gradient of Hh that define different domains of gene expression (marked by “x”). In the Overshoot model, patterning depends on a single “switching threshold,” δ, that differentiates between ON/OFF states of the pathway (see Text S1). Because of the overshoot, some cells will be exposed to the signal (above δ for a transient period of time (red solid circle), whereas others will be constantly exposed (green solid circle). Either model has the potential to explain gene expression in wild type. The dashed and solids lines represent the two different time points corresponding to the overshoot and steady-state profiles, respectively. (H) In *ptc*−TPT discs, the Hh gradient is not expected to exhibit an overshoot. The Hh gradient profiles converge to the steady-state location directly, and no posterior shift of the gradient occurs. Using the same concentration thresholds T1 and T2 defined in (G), the Classical Morphogen model predicts different Col and dppZ domains even in the absence of Ptc up-regulation. According to the Overshoot model, in contrast, no cells would be exposed to the signal only transiently and thus, an overlap of Col and dppZ patterns is predicted.

In *ptc*−TPT discs, there is no Hh-dependent *ptc* expression, and consequently, discs are not expected to exhibit a Hh overshoot. We found that the expression domains of Collier (Col) and a *dpp* reporter, dppZ, which are expressed in different domains in wild-type discs (Figure 3A–3C), are almost completely overlapping in *ptc*−TPT discs, save a single row of cells (Figure 3D–3F). This result provides insights into the mechanism of patterning, because in contrast to the wild type (Figure 3G), the Overshoot model and the Classical Morphogen model make different predictions regarding target gene expression in *ptc*−TPT discs (Figure 3H). The Classical Morphogen model predicts that these genes will continue to be differentially expressed in *ptc*−TPT discs because distinct concentration thresholds define their boundaries (Figure 3H). In contrast, the Overshoot model predicts that Col and dppZ borders would overlap in *ptc*−TPT discs; in the absence of a spatial overshoot, we predict that there are no cells that are only transiently exposed to the signal (Figure 3H and see Figure 2C).

**Figure 4:**
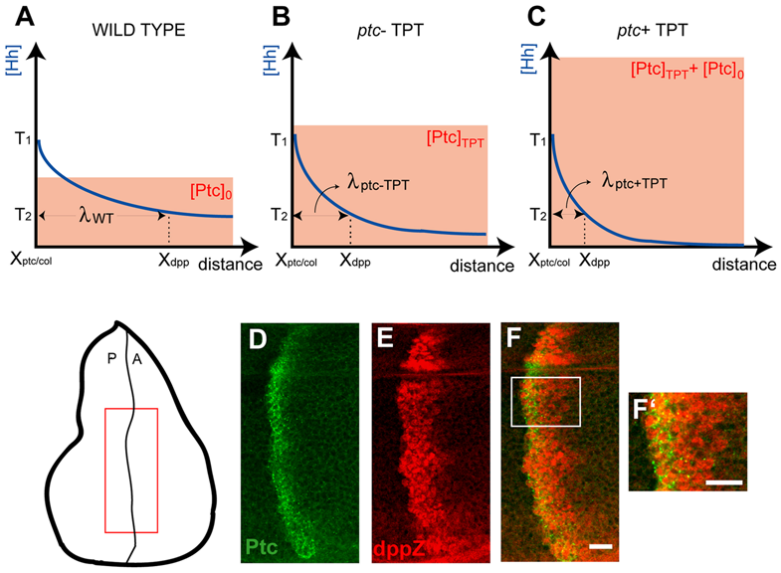
Ptc and dppZ are expressed in different domains in *ptc*+TPT discs. (A–C) Comparison of the range of the Hh gradient in wild-type (A), *ptc*−TPT (B), and *ptc*+TPT discs (C) in the region anterior to the expected *ptc* and *col* borders (X_ptc/col_). In this region, the gradients are exponential, with characteristic length given by 
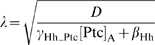
, with [Ptc]_A_ defined as the Hh-independent concentration of Ptc in the anterior compartment. On the basis of reported measurements of the TPT transgene with respect to wild type (see Text S1 [17],[18]), we assumed that [Ptc]_A_ in *ptc*−TPT discs ([Ptc]_TPT_) is higher than in wild-type discs ([Ptc]_0_). In the context of the Classical Morphogen model, the Hh gradient in (A–C) has the same amplitude (*T*_1_) at X_ptc/col_ even though that the actual value of X_ptc/col_ may be different for each case. Therefore, λ is representative of the width of the domain in which Ptc and dppZ do not overlap ([X_dpp_−X_ptc/col_]). [Ptc]_A_ should be higher in *ptc*+TPT than in *ptc*−TPT discs, and thus, λ in *ptc*+TPT disc is a lower-bound estimate of [X_dpp_−X_ptc/col_] in *ptc*−TPT discs (see Text S1). (D and E) Immunostaining of wing discs carrying the *dpp10638* reporter and a single copy of the TPT transgene using anti-Ptc (D) and anti-β-gal (E) antibodies. (F) Merge of images in (D and E). (F′) Magnification of the white box shown in (F). Scale bar indicates 10 µm.

As Ptc up-regulation also results in Hh signal inactivation, we considered the possibility that the almost overlapping patterns of Col and dppZ observed in *ptc*−TPT discs are simply a consequence of increased levels of Ptc introduced by the TPT transgene. In fact, we noted that in *ptc*−TPT discs, the predicted distance between the anterior borders of Col and dppZ [X_dpp_−X_ptc/col_], in the context of the Classical Morphogen model, is reduced with respect to the wild-type case if levels of Ptc introduced by the TPT transgene are higher than the endogenous Ptc levels expressed in wild-type discs away from the boundary (see Figure 4A and 4B and Text S1). In order to address this possibility, we considered a system in which Ptc is expressed at even higher levels than in *ptc*−TPT discs. For instance, within wild-type discs that carry a copy of the TPT transgene (*ptc*+TPT), the levels of Ptc are higher in anterior cells away from the boundary compared with *ptc*−TPT discs. If the Ptc levels introduced by the TPT were the cause of the small difference between Col and dppZ expression patterns in *ptc*−TPT discs, the prediction is that [X_dpp_−X_ptc/col_] in *ptc*+TPT discs will reduce even further (see Figure 4C and Text S1). We observed that dppZ expands at least three cells beyond the Ptc border in *ptc*+TPT discs (Figure 4D–4F). These data reveal that the overlapping patterns in *ptc*−TPT discs do not result from a dominant-negative effect of the TPT transgene and argue strongly against the Classical Morphogen model (see Discussion).

### Dynamics of Hh Target Gene Expression

In light of the Overshoot model (Figure 2C), we predicted that (1) *dpp* would respond rapidly to a transient exposure to Hh, because the transient overshoot likely occurs in a relatively short amount of time (e.g., see Figure 2F); and (2) *dpp*, but not *ptc*, expression would be predicted to persist after the transient signal is discontinued.

**Figure 5:**
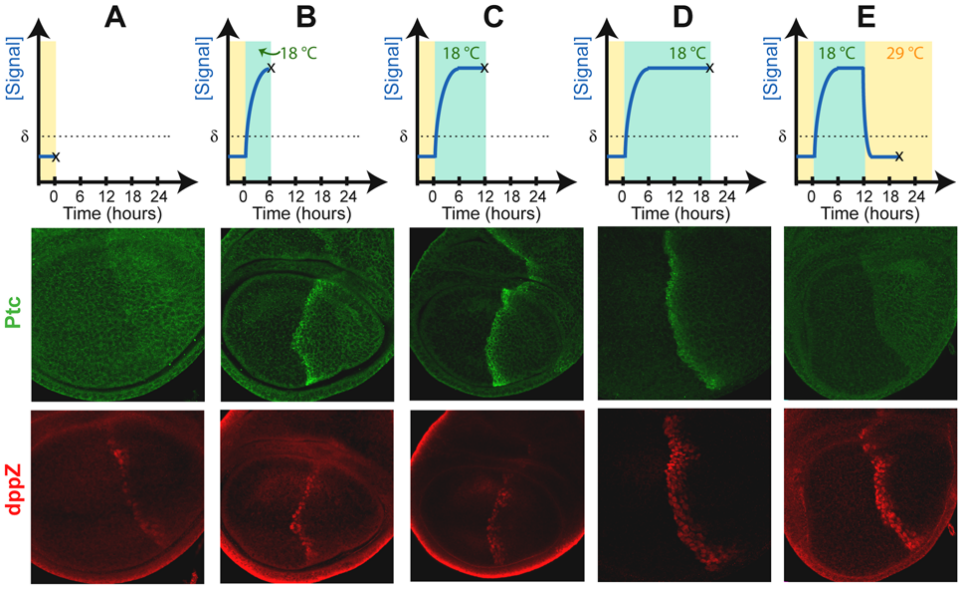
Dynamic response of Hh target genes. Third instar larvae from *hh^ts2^* homozygous animals initially grown at 18°C and obtained along a time course of Hh exposure at particular times indicated in the diagrams. The time course (also illustrated in the panels above the images) is defined by notation (*y*,*z*): homozygous animals were grown at 18°C for 8 d, followed by 48-h signal interruption at 29°C, followed by *y* hours of signal induction at 18°C, followed by an additional *z* hours of interruption at 29°C: (A) = (0,0); (B) = (6,0); (C) = (12,0); (D) = (20,0); and (E) = (12,8). In the lower panels, immunostainings depict Ptc (green) and dppZ (red) expression in discs subject to the different histories of Hh exposures as outlined in the time-course.

We investigated the response of Ptc and dppZ expression after Hh signal was restored only transiently in *hh^ts2^* homozygous discs. Consistent with previous data (Figure 2E), Ptc is fully expressed after only 6 h of signal re-initialization; in contrast, dppZ expression is limited to only one row of cells under these conditions (Figure 5B; compare to Figure 5A). The full domain of Ptc is reached after 12 h of induction (Figure 5C), but this expression can be completely eliminated after a loss of Hh activity of only 8 h (Figure 5E). In contrast, even after 12 h of induction, only partial dppZ expression appears (Figure 5C). Expression of dppZ within its entire expression domain is approached after 20 h of exposure (Figure 5D). Importantly, a similarly full dppZ expression pattern is supported when the signal is only induced for 12 h but followed by an additional interruption of 8 h (Figure 5E, compare with Figure 5D). This result suggests that additional time of exposure to the signal is not required to support the full expression of dppZ, but instead that 12 h of exposure to Hh are sufficient for approximately normal dppZ expression, but an additional waiting period is required for the pattern to fully develop (Figure 5E; compare with Figure 5C). These data also suggest that whereas sustained exposure to the signal is required to maintain Ptc expression, dppZ expression is maintained after Hh signaling has ceased (Figure 5E and see below). Collectively, these data demonstrate that *ptc* responds rapidly to changes in Hh signaling activity and suggest that although the *dpp* response is much slower, additional regulatory mechanisms may ensure its ability to capture the full dynamics of the gradient (see Discussion).

**Figure 6:**
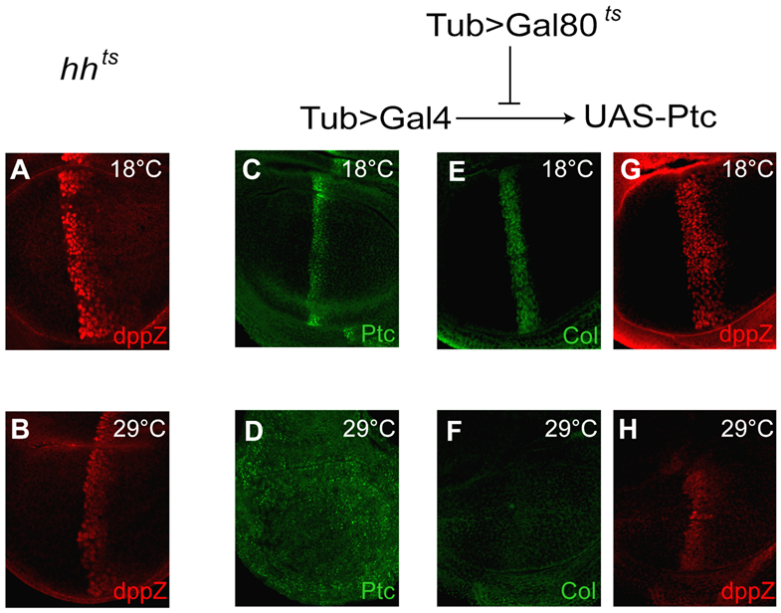
Persistence of dpp expression after Hh signaling interruption. (A and B) Antibody staining using anti-β-gal antibody to detect dppZ expression in *hh^ts2^* homozygous third instar wing discs at 18°C (A) or exposed to restrictive temperature 29°C for the last 24 h (B). The domain of dppZ expression is similar in (A and B), but the intensity of expression is higher in wild type. (C–H) Immunostaining using anti-Ptc (C and D), anti-Col (E and F), and anti-β-gal (G and H) antibodies within discs that overexpress Ptc using the Gal4-UAS system, and controlled by a temperature-sensitive Gal80. At 18°C, Gal80 inactivates ectopic Ptc expression, so discs grown at this temperature exhibit essentially normal patterns of Ptc (C), Col (E), and dppZ (G). A 24-h exposure to 29°C, however, causes high levels of Ptc expression ubiquitously (D), and consequently, the Col pattern is completely lost (F), whereas low levels of dppZ remain (H).

We investigated further the fact that dppZ expression perdures after Hh signal is interrupted using the *hh^ts2^* genetic background and found that dppZ persists after 24 h at restrictive temperature, although the intensity of expression is significantly reduced (Figure 6A and 6B). Through in situ hybridization, we confirmed that dppZ perdurance is not a consequence of β-galactosidase (β-gal) stability, as *dpp* transcript similarly persists after 24 h of signal interruption (Figure S2).

Previous studies have used Ptc overexpression to repress Hh signaling in the wing disc [30]. Using the Gal4-UAS system and a temperature-inducible Gal80 protein, we were able to interrupt Hh signaling (via strong Ptc overexpression) in a temporally controlled manner. At permissive temperature, Gal80 blocks Gal4's ability to drive Ptc expression, such that wing discs from animals developing at permissive temperature exhibit normal patterns of Ptc, Col, and dppZ (Figure 6C, 6E, and 6G). However, animals exposed to restrictive temperature for the last 24 h of larval development (Figure 6D) ectopically express Ptc ubiquitously, which abrogates Hh signaling, as confirmed by the loss of Col expression (Figure 6F). In contrast, we found that low levels of dppZ expression persist under these conditions (Figure 6H). Taken together, these data (Figures 5 and 6) suggest that whereas *ptc* (or *col*) and *dpp* expression are each initiated by Hh signaling, only *ptc* and *col* expression require sustained exposure for maintenance.

In an effort to identify the molecular machinery responsible for maintenance of *dpp* expression, we considered a role for *dpp* autoregulation. If Dpp autoregulation were responsible for maintaining expression of *dpp* in the most anterior part of its domain (that which is not overlapping with the Col/Ptc), then Ptc and dppZ would be expected to overlap in discs in which Dpp signaling is lost. To test this assertion, we used a *dpp* temperature-sensitive system (*dpp^ts^*) [31]. *dpp^ts^* discs develop normally at permissive temperature; in particular, they have normal patterns of phosphorylated Mad (pMAD), a direct indicator of Dpp signaling activity, as well as normal expression of Ptc and dppZ (Figure S3A–S3C). However, *dpp^ts^* discs, exposed to restrictive temperature for the last 24 h of larval development, exhibit a loss of pMAD expression (Figure S3E), but Ptc and dppZ genes are expressed in their normal domains (Figure S3F and S3G); in particular, these patterns do not overlap (Figure S3H). Thus, this result argues against the idea that Dpp autoregulation is the main mechanism that controls *dpp* maintenance.

## Discussion

In this study, we investigated how the Hh gradient is interpreted in the *Drosophila* wing imaginal disc. In particular, we explored whether or not concentration thresholds in the Hh gradient correspond to different borders of target gene expression. We used mathematical modeling as a hypotheses-generating tool. Although our mathematical model makes strong claims about the interpretation of Hh signaling, it is through in vivo experimentation that these hypotheses were tested. The predictions of the mathematical analysis present a novel mechanism for Hh signal interpretation by a field of cells. This model can be summarized by three main claims (see Figure 2C): (1) At steady state, a monotonic Hh gradient is translated into a step-like signal response. This suggests that only two states (“blue”/“red,” corresponding to fully ON/OFF expression of Hh target genes) can be discriminated at steady state. (2) The dynamics of the Hh gradient (and therefore the signal response) exhibit a spatial overshoot. This raises the possibility that a third state (“white,” corresponding to *dpp* ON; *ptc*/*col* OFF expression) may be established by the transient signal provided by the overshoot. (3) This third state (white) may require additional mechanisms to sustain gene expression after the transient exposure to Hh ceases. Here, we discuss these predictions in the light of our experimental data.

### Existence of a Hh Overshoot and Dynamics of Target Gene Expression

We provide in vivo evidence that Hh signaling responds to dynamical changes of the gradient. Upon Hh signaling reinitialization (using the *hh^ts2^* system), the establishment of the Hh gradient is dynamic and exhibits an “overshoot behavior” (Figure 2E and 2F). Although the existence of the Hh overshoot can be predicted from the gene network architecture, it was not clear if the timescale of gradient formation would be slow enough such that the overshoot could be detected at the level of target gene expression. Using Ptc expression as a reporter of Hh signaling activity, we demonstrated that a Hh overshoot can influence Hh-dependent patterning. Ptc is fully up-regulated within 6–9 h of reinitialization of the system (Figure 2E), therefore, we estimate that the transient overshoot occurs on a similar timescale; in contrast, refinement of the gradient likely happens at a much slower pace (Figure 2F). Furthermore, it is not clear from our data whether the refinement occurs progressively (as predicted in Figure 2B) or in a stepwise manner (for example, oscillating towards a final state), and either scenario would be consistent with an Overshoot model.

However, the natural timing of an overshoot during the course of normal development remains in question. Because *hh* is expressed in the early embryo, one possibility is that the Hh gradient forms early during embryonic development and is retained within the wing disc. Of note, however, is the fact that several factors are required for Hh secretion and distribution [32]–[35]. As it remains unclear at which developmental time point Hh mobility might be afforded by one or more of these factors, it is difficult to speculate on the timing of an overshoot. In the future, live examination of the distribution of Hh from early larval development and throughout maturation of wing disc development will provide insights, but currently, this remains a technical challenge.

Our data also revealed that *ptc* expression is up-regulated faster than *dpp* expression (Figure 5). Moreover, the temporal response of *dpp* expression in response to Hh signaling (12–20 h) is much slower that the predicted timescale of the occurrence of the overshoot (6–9 h). This result is counterintuitive with regards to the Overshoot model because it predicts that the domain of *dpp* expression can be specified by a transient Hh signal. Interestingly, our data demonstrate that only a short transient exposure to Hh appears to be required to support *dpp* expression, but it can only be detected some time thereafter (see Figure 5). Thus, additional regulation independent of Hh is likely to influence the timing of gene expression. For example, the kinetics of *dpp* expression may be controlled by a feed-forward loop in which a factor required for its activation introduces a time delay with respect to the time in which exposure to the Hh signal takes place (see below).

### Signal-Dependent Ptc Up-Regulation Is Required to Generate Multiple Patterns

Our in vivo data demonstrated that in the absence of Hh-dependent Ptc up-regulation, the borders of Col and dppZ coincide (Figure 3F). We contend that this result is not due to higher levels of Ptc supported by the TPT transgene, because wild-type discs expressing this same TPT transgene (*ptc*+TPT discs) should express even higher concentrations of Ptc in the anterior compartment (due to additional contributions from the endogenous *ptc* gene), and yet these discs clearly exhibit different domains of Ptc and dppZ expression (Figure 4). Thus, the Classical Morphogen model cannot explain the patterns observed in *ptc*−TPT and *ptc*+TPT discs. Importantly, the nonoverlapping domains of Ptc and dppZ in *ptc*+TPT (Figure 4D–4F) are consistent with the Overshoot model, as Hh-dependent Ptc up-regulation is supported within these discs.

Nonetheless, other interpretations to explain the overlapping patterns in *ptc*−TPT discs are plausible. For example, we observed that whereas the Col domain of expression expands in *ptc*−TPT discs relative to wild-type discs (Figure 3D, compare with Figure 3A), the dppZ expression domain does not (Figure 3B and 3E). It is formally possible that the Col and dppZ borders may coincide in the *ptc*−TPT discs due to a postulated repressor that blocks Hh target gene expression beyond a certain position within the anterior compartment. However, we suggest this scenario is unlikely, as a significant expansion of the dppZ domain is observable both in *ptc* mutant clones located near the AP boundary or when lower levels of Ptc are present [18]. Furthermore, Hh-expressing clones located anywhere in the anterior compartment are able to support expression of target genes within and around the clone [6].

Another possible interpretation is provided by a recent study of Hh signaling in the vertebrate spinal cord in which signal-dependent Ptc up-regulation provides cells the ability to adapt after sustained exposure to the Hh signal (“desensitization”) [36]. This “Temporal Adaptation model” also invokes feedback by up-regulated Ptc; however, the role of the Ptc protein in this model is quite different. The Temporal Adaptation model relies on Ptc to down-regulate the signaling pathway; in contrast, the Overshoot model depends on the ability of Ptc to sequester the ligand. Supposing that desensitization is in effect, Ptc up-regulation could convert different Hh concentrations into a more graded signal response (Figure S4A), but such an effect would be limited to the domains in which Ptc is expressed, as desensitization is cell autonomous in nature. Thus, cells that express dppZ (but not Col) should not be affected by Ptc-dependent desensitization (Figure S4B). We argue, therefore, that the Temporal Adaptation model cannot explain the patterning changes we observe in *ptc*−TPT discs, i.e., the overlap between *dpp* and *ptc/col* expression domains (see Figure S4). Alternatively, *en* is considered responsive to the highest levels of Hh signaling (see Figure 1A) [6],[37],[38], and En and Col overlap in expression within *ptc*−TPT discs (Figure S5). However, we cannot be sure that this is due to loss of desensitization, because a careful examination of En expression in wild-type discs shows that Col and En in fact overlap even in wild-type discs, provided that gene expression is assayed late enough in development (Figure S6). Nevertheless, it is still plausible that Hh-dependent Ptc up-regulation may control the boundary position of other Hh target genes in two ways: by Ptc-dependent desensitization within the domain of *ptc* expression and through gradient dynamics (overshoot) in cells located anteriorly to the *ptc* domain.

Although the Overshoot model is largely consistent with our observations, the fact that Col and dppZ patterns in *ptc*−TPT discs do not completely overlap (Figure 3F′) requires additional discussion. Because the Overshoot model strictly assumes a unique threshold, δ, that distinguishes between ON and OFF states of the system (Figure 2C), in theory, the model would predict a full overlap of Col and dppZ within *ptc*−TPT discs. Though we contend that gradient dynamics, driven by signal-dependent Ptc up-regulation, encodes the predominant mechanism by which gene expression boundaries are established, additional mechanisms could also subtly influence patterning outputs. For instance, interpretation of the ON/OFF threshold at the *cis*-regulatory level cannot be infinitely accurate, and thus, different binding affinities (i.e., concentration-dependent effects) might account for small differences in the observed expression domains (Figure 3F).

### A State-Space Model for the Overshoot Model of Patterning

**Figure 7:**
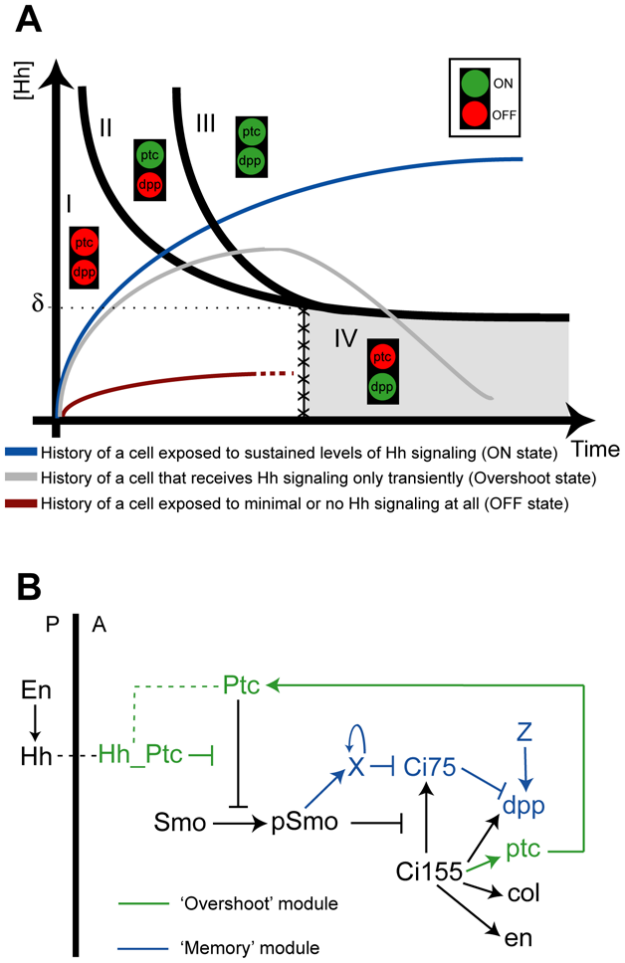
State-space diagram and network architecture for the Overshoot model. (A) The state-space diagram consistent with the Overshoot model is divided into four territories (I–IV) defined by the expression levels of *ptc* (or *col*) and *dpp*, consistent with the data in Figure 5. All cells in the anterior compartment are initially in Territory I (*ptc* OFF; *dpp* OFF), but only cells relatively far from the AP boundary, in which signaling levels are below the switching threshold, δ, remain in this territory (red trajectory). During the formation of the Hh gradient, cells located sufficiently close to the AP boundary visit transiently Territory II and express *ptc*, but not *dpp*. From the subset of cells that enter Territory II, those that remain exposed to the signal will continue expressing *ptc* and will eventually express *dpp* when crossing to Territory III (blue trajectory). However, cells in which Hh signaling ceases (for example, as a result of gradient refinement after the overshoot) will maintain *dpp* expression, but will stop expressing *ptc*. These cells will cross to Territory IV and remain there (gray trajectory). Note that Territory IV is not simply connected to Territory I (see boundary line demarcated by X's) as it is only accessible from Territory III. An additional territory (V) may be considered to include *engrailed* in the state-space model (see Figure S6G). (B) The Overshoot model is inherent within the Hh network architecture. The overshoot of Hh depends exclusively on Hh-dependent Ptc up-regulation (“Overshoot” module). However, distinct *ptc/col* and *dpp* can only be realized if *dpp* (but not *ptc* and *col*) expression is maintained in cells in which exposure of Hh is only transient. One way in which this can be effected at the molecular level is if a “Memory” module operates in the network. For example, *dpp* expression can be maintained specifically if a stable protease “X” that degrades the repressor form of Ci (Ci75) is up-regulated in response to Hh signaling; “Z” represents a Hh-independent activator that supports *dpp* expression (see Discussion).

Our results can be summarized by a state-space diagram, which integrates dose-dependent effects with gradient formation dynamics (Figure 7A). The history of Hh signaling activity over time for a given cell is represented by a trajectory within the state-space diagram. Cells in the anterior compartment adopt one of *three* final territories (I, III, and IV) depending on their history of Hh exposure rather than their final Hh concentration. Territory II is a transient state in which the cell expresses *ptc*, but not *dpp*. An unusual topological property of this state-space diagram is that Territory IV can only be reached through Territory III. A further requirement is that cells that enter Territory IV be able to maintain *dpp* expression, for instance through a positive feedback loop that allows cells to retain *dpp* expression even once Hh signaling has been discontinued. In support of this requirement, our data show that *dpp* expression is maintained long after Hh signaling is interrupted (Figure 6).

Autoregulatory feedback is an attractive hypothesis to explain this maintenance of *dpp* expression, as previous studies suggest *dpp* exhibits autoregulation in the wing disc [39] and in the early embryo [40],[41]. However, we see no change in the dppZ pattern when Dpp signaling is transiently impaired (Figure S3), arguing against autoregulatory feedback as a major mechanism supporting “memory” of *dpp* expression. However, other possible molecular mechanisms to maintain *dpp* expression are conceivable (see below and Figure 7B, for example).

### Towards the Molecular Basis of the Overshoot Model

Our data are consistent with a model in which Hh-dependent Ptc up-regulation is necessary to shift the initial Hh gradient posteriorly, generating a zone of transient signal exposure that is required to specify different domains of gene expression. Unlike other models of patterning, the Overshoot model is based on two properties of the Hh gene network architecture: first, a negative feedback loop mediated by Ptc that controls the spatial range of the Hh gradient (Figure 7B; “Overshoot” module) [18], and second, a positive feedback loop that maintains gene expression of *dpp* in the region in which only a transient Hh signal is received (Figure 7B; “Memory” module).

Although we contend that the molecular players within the Overshoot module are known, we do not have a molecular understanding of the genes that relate to the Memory module. One possible molecular mechanism to explain “memory” may involve the differential functions of Ci. *ptc*, *col*, and *en* require the activator form of Ci (Ci155) for expression; whereas in the absence of Ci155, *dpp* still exhibits low-level expression [42]. Evidence exists that the repressor form of Ci (Ci75) is more critical for *dpp* expression and that another activator “Z” may support low level *dpp* expression [42]. A putative Memory module, for example, could involve Hh-dependent regulation of a protease, “X,” that degrades Ci75 and is stable. In this scenario, the overshoot gradient would be sufficient to activate X in the *dpp* expression domain, which in turn will maintain the absence of Ci75 in this region even after the overshoot has occurred. This molecular model is appealing in light of our data for the following reasons. First, it suggests that the slow temporal response of the *dpp* pattern after signal reinitialization may be due to the delay in producing X; and second, it explains why *dpp* can only be maintained at low levels after Hh signaling is removed (Figure 6 and Figure S2).

### Implications of the Overshoot Model

We have discussed how the Overshoot model proposed here explains *ptc/col* and *dpp* differential expression, but how might expression of *en* be controlled? We observed that the En boundary falls in the middle of the Ptc domain in the wing disc of a crawling third instar larva (Figure S6A–S6C), but the patterns tend to overlap when discs are taken from larvae close to pupariation (Figure S6D–S6F). These results suggest that the En pattern forms very slowly and may also be explained in the context of our three-state overshoot model: En is patterned in a manner similar to *ptc* and *col* genes, encompassing similar domains of expression, but the En pattern is realized later (Figure S6G).

These observations raise an important point regarding the interplay of gradient dynamics and Hh concentration levels. Although the Overshoot model does not require multiple concentration thresholds in order to determine different boundaries of gene expression, the rate of signal activation likely depends on the Hh concentration. The influence of concentration on patterning through the Overshoot model is explicitly taken into account within the state-space diagram, because different territories are delineated by curved domains (instead of straight vertical lines) to indicate responsiveness to concentration differences (Figure 7A and Figure S6G). For example, cells exposed to two different Hh concentrations (provided that they are both higher than the switching threshold, δ) will both eventually turn *en* expression ON, but the cell exposed to the higher concentration will support expression first. This would also explain why the dppZ pattern forms in a sequential manner, with fewer cells closer to the AP boundary expressing the reporter first (Figure 5). To be clear, this concentration-dependent influence on the rate of pattern formation is *not* related to concentration thresholds as defined by the Classical Morphogen model.

Another implication of the Overshoot model is that the decision to activate *dpp* is irreversible. This implies that once cells receive sufficient Hh levels to turn on the pathway and express *dpp*, they will continue to do so even if Hh signal is removed (Figure 6). This idea is similar to early theoretical studies based on the classical French Flag model that proposed that the ability to reach a certain concentration threshold is not sufficient to support a specific response, but that instead, additional feedback interactions are required to “lock down” that response [43],[44]. We find support for this idea as we observe that *dpp* expression is retained even 24 h after Hh protein is removed (Figure 6B and Figure S2).

Although the Overshoot model as presented here (Figure 7A) can only specify three states (i.e., a “French Flag”), it is conceivable that this model may be generalizable to support more than three distinct domains of expression. For example, an additional state in the system may be incorporated if additional proteins are considered, ones that, like Ptc, regulate Hh mobility and are up-regulated in response to the signal. In vertebrates, targets of Hh have been identified that include proteins that sequester Hh itself. Hedgehog-interacting protein 1 (Hhip1) can limit the range of the Hh gradient, and *Hhip1* transcription is also slowly up-regulated by Hh signaling [45]. In this case, for instance, the full refinement of the Hh gradient could occur in two discrete steps (in vertebrates, this could be mediated by Ptch1 and a Hhip1) that generate two transient zones of different temporal exposures to Hh able to support multiple patterns of gene expression. In fact, in *Ptch1* mutant mouse embryos that express low levels of Ptch1 ubiquitously, neural tube patterning is affected in ventral regions (close to the source of Hh), but patterning of intermediate regions is approximately normal [46]. However, in *Ptch1*; *Hhip1* double mutants, ventral patterns expand and overlap intermediate patterns [46]. These observed patterning changes share similarity with the overlap of patterns observed for *ptc*−TPT discs (Figure 3D–3F); thus, we suggest that the Overshoot model may apply to Hh patterning in vertebrates as well.

### Conclusion

The significance of the Overshoot model presented here relies on the architecture of a particular gene regulatory network, in which a morphogen activates the expression of a molecule affecting its distribution. As this network property has been identified in other systems [45],[47]–[49], it is possible that evolution has selected upon this network architecture to support patterning of other developing systems in a similar manner to Hh-mediated patterning of the *Drosophila* wing disc, through gradient dynamics.

## Materials and Methods

### Numerical Simulations

A Forward-in-Time-Centered-in-Space (FTCS) algorithm (Δ*x* = 2.5 µm, Δ*t* = 0.5 s) was implemented to solve Equations 1–5 numerically in MATLAB using the parameters in Table S1. Numerical solutions approximately reach steady state in less than 8 h (see Text S1). To simulate the outputs in *ptc*−TPT (Figure 3H), we use Equation S4 instead of Equation 2 (see Text S1 for further details).

### Fly Crosses and Transgenes

Fly crosses were conducted at 25°C, except where otherwise indicated. For the experiments using the *hh^ts2^* allele (Figures 2E, 5, 6A, and 6B, and Figure S2), fly stocks of genotype *dpp10638*/CyO; *hh^ts2^*/TM6B, Tb were crossed at 18°C. *dpp10638* is a transgene on II containing a lacZ reporter (dppZ) that produces nuclear β-gal. *hh^ts2^* homozygous animals are marked by the Tb^+^ phenotype. A Tubulin1>*ptc*>Tubulin1α 3′UTR (TPT) transgenic line located on chromosome III has previously been shown to rescue *ptc* mutant animals [18]. To obtain *ptc*−TPT discs, we crossed *ptc^9^*;TPT/SM6; TM6B, Tb males to *ptc^16^*, dpp10638; +/SM6; TM6B, Tb females. *ptc^16^* has been previously characterized as a null allele [50], and *ptc^9^* produces a product that is defective in reaching the plasma membrane and binding to Hh [51]. Tb^+^ marks *ptc* mutant larvae that carry a copy of both the TPT and *dpp10638* transgenes (Figure 3D–3F and Figure S5). In Figure 4D–4F, TPT transgenic discs in a wild-type background (*ptc*+TPT discs) were obtained from third instar larvae of the genotype *dpp10638*; TPT/SM6; TM6B, Tb. In Figure 6C–6H, males of genotype *dpp10638*; UAS-Ptc/SM6; TM6B, Tb were crossed to females of genotype TubGal80^ts^; TubGal4/SM6; TM6B, Tb at 18°C. TubGal80*^ts^* is a transgene on chromosome II that expresses a temperature-sensitive form of the Gal80 protein. In these experiments, third instar larvae of Tb^+^ phenotype were selected, representing mutants of genotype TubGal80^ts^/*dpp10638*; TubGal4/UAS-Ptc. *dpp^hr56^*/*dpp^hr4^* transheterozygote animals are defective in Dpp signaling at 29°C [31]. We crossed *dpp^hr56^*; +/SM6; TM6B, Tb to *dpp^hr56^*; dppZ/SM6; TM6B, Tb animals at 18°C. Third instar larvae marked by Tb^+^ are normal at 18°C but defective in Dpp signaling after 24 h at 29°C (Figure S3).

### Reinitialization of the Hh Gradient Using a Temperature-Sensitive *hedgehog* Allele

*hh^ts2^* flies were placed at 18°C. After 2 d, adults were removed and progeny were kept at 18°C for an additional 7 d. Hh signaling was then interrupted by placing the resulting larvae at 29°C for 24 h. Finally, larvae were placed back at 18°C to induce Hh signaling for a period of time, τ. Third instar larvae homozygous for the *hh^ts^* allele were fixed immediately for immunostaining at the designated time points τ. A graphical depiction of this scheme is presented in Figure 2D.

### Fixation, In Situ Hybridization, and Immunostaining

Third instar wing discs were dissected, fixed, and immunostained using standard techniques. The following primary antibodies were used: monoclonal mouse anti-Ptc (developed by I. Guerrero, and was obtained from the Developmental Studies Hybridoma Bank at the University of Iowa), rabbit anti-β-gal (Invitrogen Molecular Probes), monoclonal mouse anti-Col (M. Crozatier), rabbit anti-En (P. O'Farrell), and guinea pig anti-pMad (E. Laufer). The secondary antibodies used were rabbit Alexa 647, mouse Alexa 488, and guinea pig Alexa 555 (Molecular Probes). Samples were mounted in Mowiol 4–88 (Molecular Biosciences). Fluorescent in situ hybridization for data in Figure S2 was performed using standard techniques using a digoxigenin-labeled riboprobe for *dpp*. Primary and secondary detection of the *dpp* probe was done using a polyclonal sheep anti-digoxigenin (Roche) and sheep Alexa 555 (Molecular Probes) antibodies, respectively.

### Image Analysis

Images were acquired using a confocal microscope (Zeiss Pascal) and processed in Adobe Photoshop. Images in Figure S2 were taken using a 20× objective (Zeiss). Images in other figures and supporting figures were taken using a 40× oil objective (Zeiss). Imaging parameters for each set of data were selected for the control or wild-type experiment, and the same imaging conditions were used throughout the rest of each dataset. Discs compared in Figure 2E were chosen such that they meet the following conditions: (1) approximately the same size, (2) similar average background intensity in the posterior compartment, and (3) similar average intensity in the anterior compartment away from the AP boundary. For the intensity profiles in Figure 2E, images were processed in ImageJ and quantified in MATLAB (see Protocol S1 for further details).

## Supporting Information

Figure S1 **Temperature changes do not affect Ptc expression.** Wild-type discs from larvae raised at 18°C (A) or from larvae raised at 18°C followed by 24 h at 29°C (B) immunolabeled for Ptc. Fixation, immunostaining, and imaging of discs in (A and B) were performed under identical conditions.
(1.39 MB TIF)

Figure S2 **dpp expression is maintained after Hh signaling is interrupted.** In situ hybridization using a riboprobe to *dpp* in a wild-type disc (A) versus a *hh^ts2^* homozygous disc (B) grown at 18°C and exposed to 29°C for the last 24 h of the third larval instar. The domain of *dpp* expression is similar in (A and B), but the intensity of expression is higher in wild type. If residual Hh levels were to account for this expression, then *dpp* expression domain would be predicted to shift in expression toward the AP boundary; the full extent of the pattern would not be expected.
(0.91 MB TIF)

Figure S3 **dpp and ptc expression is normal after Dpp signaling interruption.** (A–C) *dpp^hr5^* ^6^/*dpp^hr^* ^4^ animals raised at 18°C are normal in Dpp signal transduction assayed by pMAD expression (A) and have normal patterns of Ptc (B) and dppZ (C). (D) Merge of the patterns displayed in (B and C). (E–G) *dpp^hr5^* ^6^/*dpp^hr^* ^4^ larvae exposed to restrictive temperature (29°C) for 24 h have lost their pMAD expression pattern (E), and yet, *ptc* and *dpp* are approximately normal (F and G). The patterns do not overlap, suggesting that Dpp signaling is not required for maintenance of *dpp* expression in the nonoverlapping region. (H) Merge of the patterns displayed in (F and G). In this figure, the dppZ transgene is an insertion on chromosome III, to allow assay in a *dpp* mutant background.
(6.07 MB TIF)

Figure S4 **The overlap of Col and dppZ in** ***ptc*** **−TPT discs cannot be explained by the Temporal Adaptation model.** Predictions of Hh patterning in wild-type (A) versus *ptc*−TPT discs (B) according to the Temporal Adaptation model [36]. In wild-type discs (A), Ptc-mediated desensitization is required to map different concentrations of the extracellular gradient (green) into a graded signal response (blue). However, when signal-mediated Ptc up-regulation is impaired, cells are unable to differentially “desensitize” the levels of the signal and respond similarly to different concentrations of the signaling (B). Thus, lack of desensitization in *ptc*−TPT discs results in the expansion of the highest response (e.g., *en*; blue) to the extent of the intermediate response (e.g., *col*; white), but should have little or no effect in the differential establishment of the dppZ and Col borders, because Ptc-mediated desensitization is a cell-autonomous effect.
(7.93 MB TIF)

Figure S5 **Col and the anterior pattern of En overlap in** ***ptc*** **−TPT discs.** Col (A) and En (B) are expressed in nearly the same domain in the anterior compartment in late third instar *ptc*−TPT discs. (C) Merge of panels displayed in (A and B). The line drawn from the Col pattern shows that Col and En approximately share their anterior border.
(2.96 MB TIF)

Figure S6 **Hh-dependent expression of Ptc and En approximately overlap in late wild-type discs.** (A and B) Immunostaining of wild-type wing discs from a crawling third instar larva using anti-En (A) and anti-Ptc (B) antibodies. (C) Merge of images in (A and B). (A′–C′) 4× magnification of the white box depicted in (A–C). White circles mark a cell in the anterior border of the En pattern showing that at this time, the En border approximately falls within the domain of Ptc expression, but does not share the same anterior boundary. (D and E) Same as (A and B), but from a third larva close to pupariation. (F) Merge of images in (D and E). (D′–F′) Magnification of the white box in (D–F). Scale bars indicate 10 µm. White circles mark a cell at the anterior boundary of the En pattern, showing that at this time, the En and Ptc anterior borders coincide. (G) Generalization of the state-space model in Figure 5 to incorporate *engrailed* (*en*). No additional concentration threshold is required to define the *en* domain of expression. Instead, *en* seems to be responsive to integration of Hh signaling over time, as it shares an anterior boundary with Ptc, and presumably Col, at later time points. Therefore, cells exposed to two different Hedgehog concentrations δ_1_ and δ_2_ above the switching threshold, δ, turn on Hh target gene expression at different time points but eventually activate all target genes (Territory V).
(2.88 MB TIF)

Protocol S1 **Generation of intensity profiles for** **Figure 2E** **.**
(0.03 MB DOC)

Table S1 **Parameter values used in the computer simulations.**
(0.09 MB DOC)

Text S1 **Supporting Text and References.**
(0.31 MB DOC)

## Abbreviations

AP: anterior–posterior
