# Supplementary figures and images for: Dynamic Interpretation of Hedgehog Signaling in the Drosophila Wing Disc
Source: PLoS Biol. 2009 Sep 29;7(9):e1000202. doi: 10.1371/journal.pbio.1000202 (PMC2744877; doi:10.1371/journal.pbio.1000202)

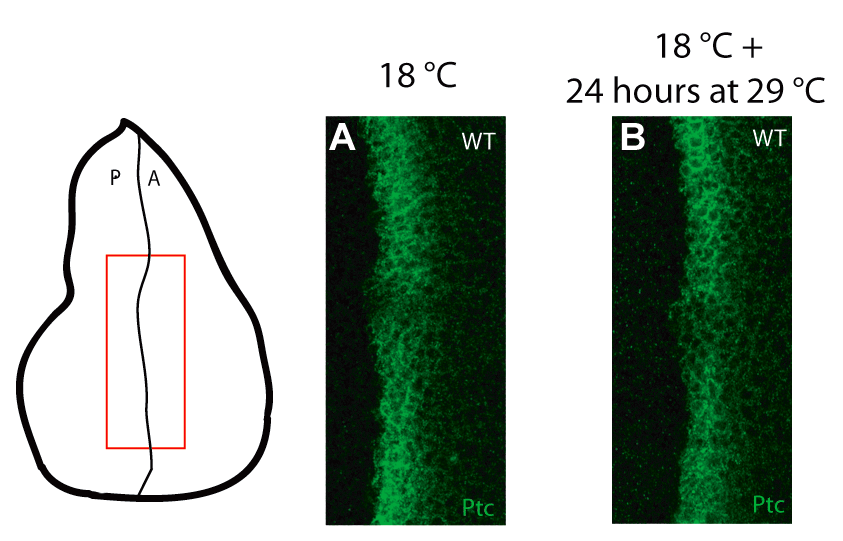

Supplement: Figure S1 — Temperature changes do not affect Ptc expression. Wild-type discs from larvae raised at 18°C (A) or from larvae raised at 18°C followed by 24 h at 29°C (B) immunolabeled for Ptc. Fixation, immunostaining, and imaging of discs in (A and B) were performed under identical conditions. (1.39 MB TIF) [file pbio.1000202.s001.tif]

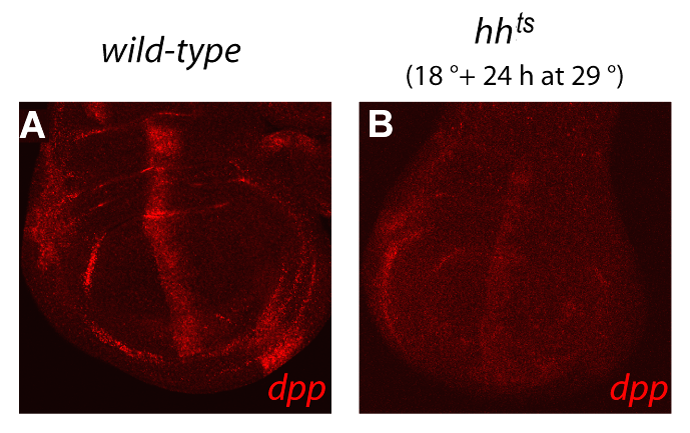

Supplement: Figure S2 — dpp expression is maintained after Hh signaling is interrupted. In situ hybridization using a riboprobe to dpp in a wild-type disc (A) versus a hhts2 homozygous disc (B) grown at 18°C and exposed to 29°C for the last 24 h of the third larval instar. The domain of dpp expression is similar in (A and B), but the intensity of expression is higher in wild type. If residual Hh levels were to account for this expression, then dpp expression domain would be predicted to shift in expression toward the AP boundary; the full extent of the pattern would not be expected. (0.91 MB TIF) [file pbio.1000202.s002.tif]

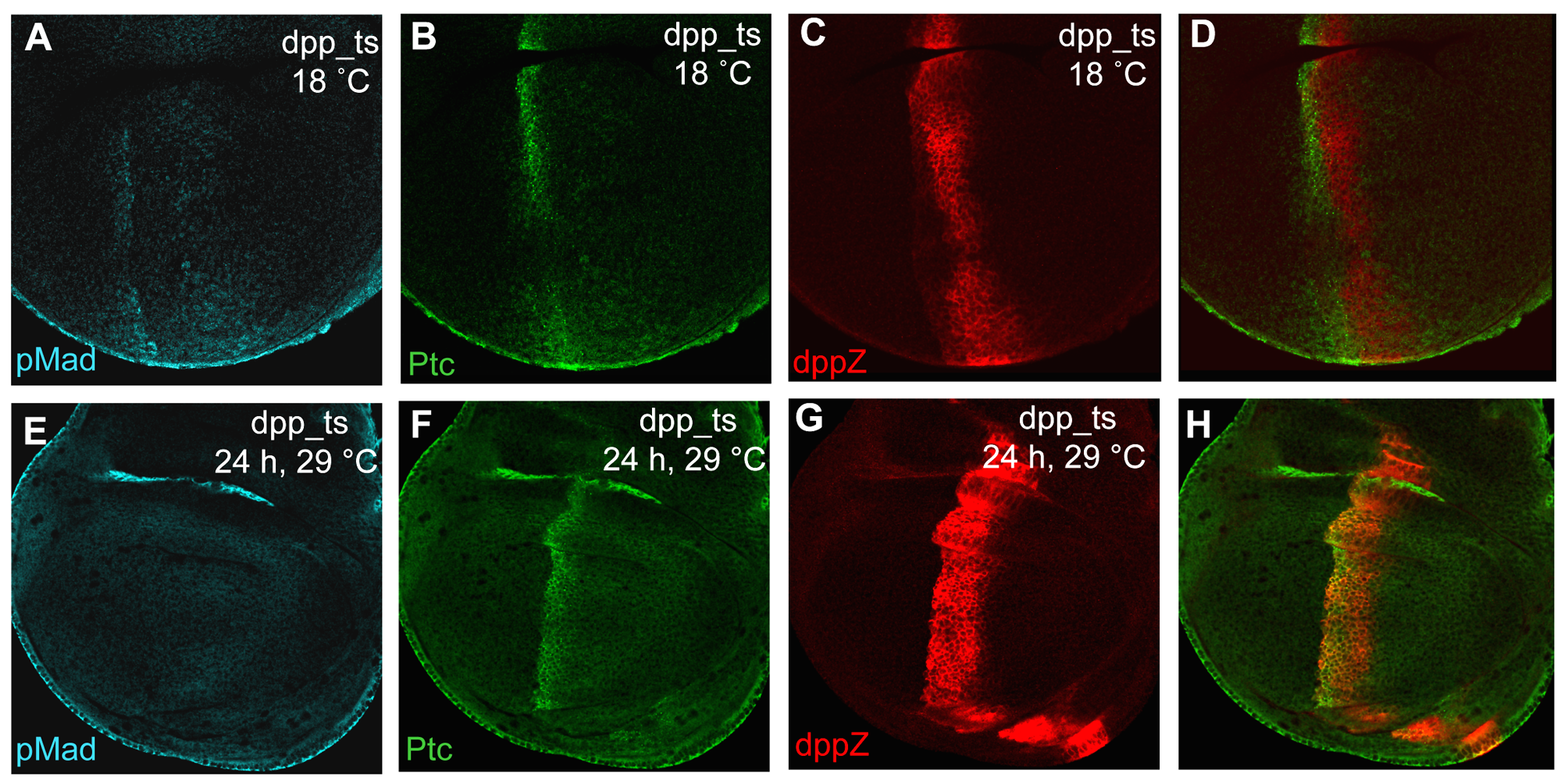

Supplement: Figure S3 — dpp and ptc expression is normal after Dpp signaling interruption. (A–C) dpphr5 6/dpphr 4 animals raised at 18°C are normal in Dpp signal transduction assayed by pMAD expression (A) and have normal patterns of Ptc (B) and dppZ (C). (D) Merge of the patterns displayed in (B and C). (E–G) dpphr5 6/dpphr 4 larvae exposed to restrictive temperature (29°C) for 24 h have lost their pMAD expression pattern (E), and yet, ptc and dpp are approximately normal (F and G). The patterns do not overlap, suggesting that Dpp signaling is not required for maintenance of dpp expression in the nonoverlapping region. (H) Merge of the patterns displayed in (F and G). In this figure, the dppZ transgene is an insertion on chromosome III, to allow assay in a dpp mutant background. (6.07 MB TIF) [file pbio.1000202.s003.tif]

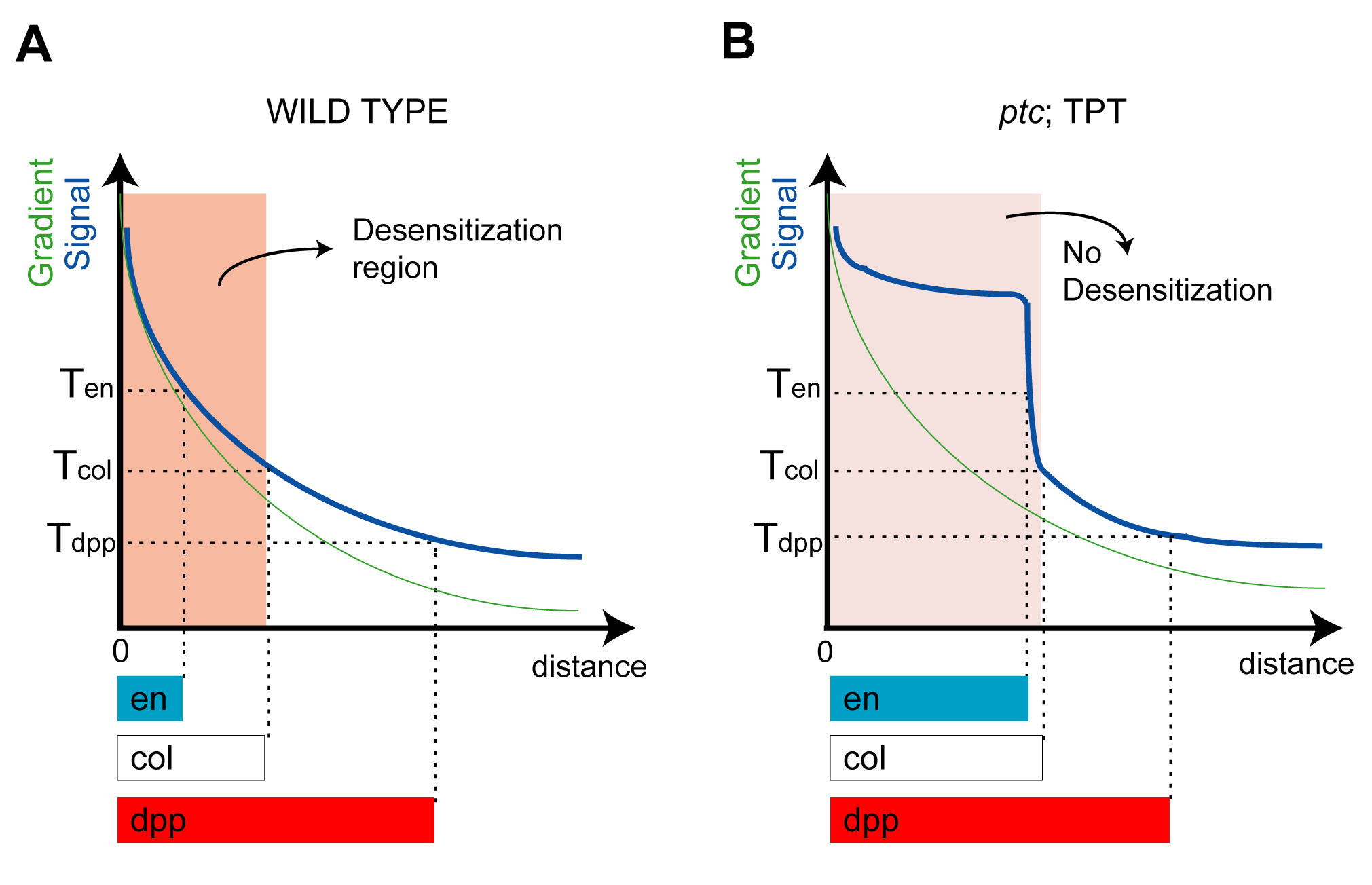

Supplement: Figure S4 — The overlap of Col and dppZ in ptc −TPT discs cannot be explained by the Temporal Adaptation model. Predictions of Hh patterning in wild-type (A) versus ptc−TPT discs (B) according to the Temporal Adaptation model [36]. In wild-type discs (A), Ptc-mediated desensitization is required to map different concentrations of the extracellular gradient (green) into a graded signal response (blue). However, when signal-mediated Ptc up-regulation is impaired, cells are unable to differentially “desensitize” the levels of the signal and respond similarly to different concentrations of the signaling (B). Thus, lack of desensitization in ptc−TPT discs results in the expansion of the highest response (e.g., en; blue) to the extent of the intermediate response (e.g., col; white), but should have little or no effect in the differential establishment of the dppZ and Col borders, because Ptc-mediated desensitization is a cell-autonomous effect. (7.93 MB TIF) [file pbio.1000202.s004.tif]

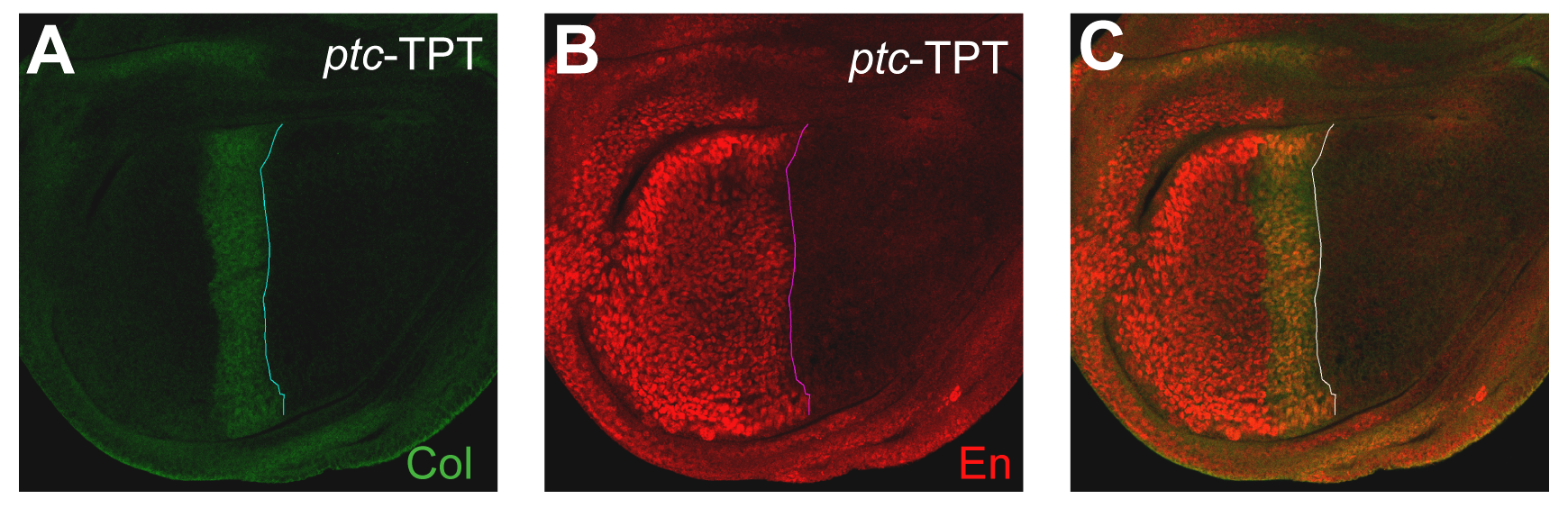

Supplement: Figure S5 — Col and the anterior pattern of En overlap in ptc −TPT discs. Col (A) and En (B) are expressed in nearly the same domain in the anterior compartment in late third instar ptc−TPT discs. (C) Merge of panels displayed in (A and B). The line drawn from the Col pattern shows that Col and En approximately share their anterior border. (2.96 MB TIF) [file pbio.1000202.s005.tif]

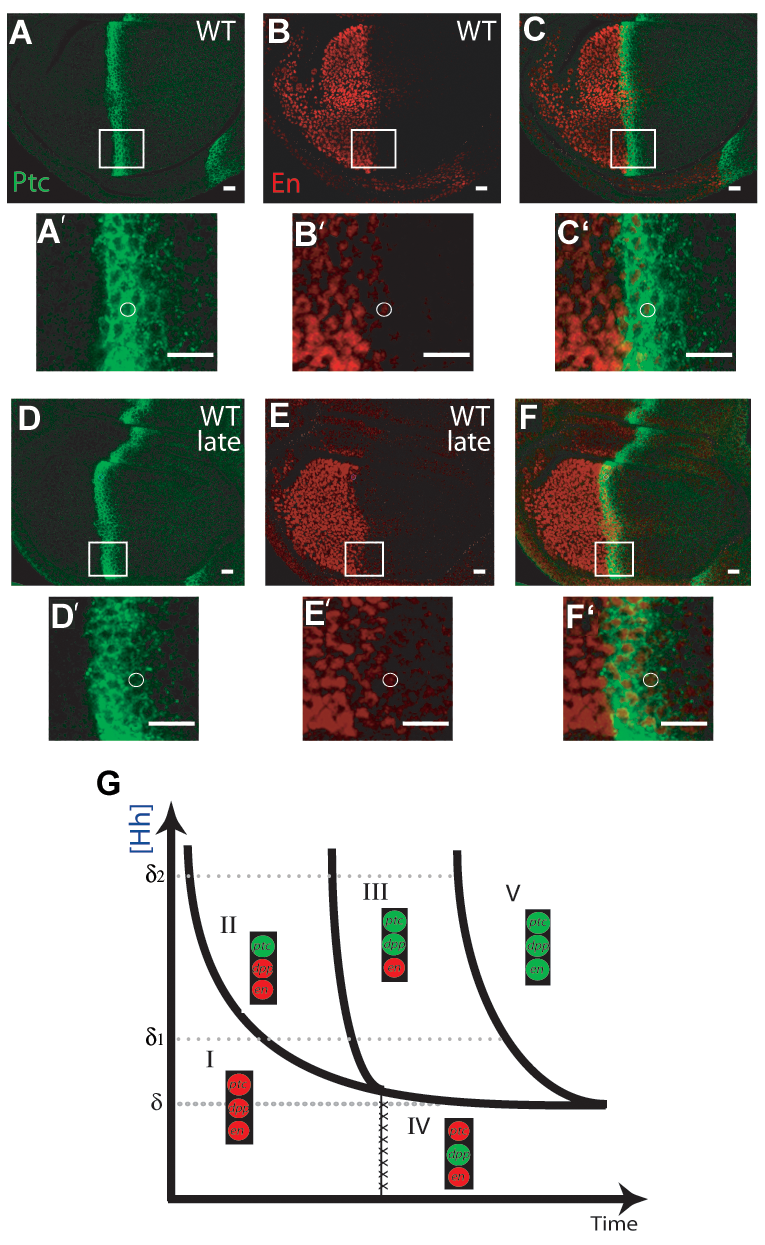

Supplement: Figure S6 — Hh-dependent expression of Ptc and En approximately overlap in late wild-type discs. (A and B) Immunostaining of wild-type wing discs from a crawling third instar larva using anti-En (A) and anti-Ptc (B) antibodies. (C) Merge of images in (A and B). (A′–C′) 4× magnification of the white box depicted in (A–C). White circles mark a cell in the anterior border of the En pattern showing that at this time, the En border approximately falls within the domain of Ptc expression, but does not share the same anterior boundary. (D and E) Same as (A and B), but from a third larva close to pupariation. (F) Merge of images in (D and E). (D′–F′) Magnification of the white box in (D–F). Scale bars indicate 10 µm. White circles mark a cell at the anterior boundary of the En pattern, showing that at this time, the En and Ptc anterior borders coincide. (G) Generalization of the state-space model in Figure 5 to incorporate engrailed (en). No additional concentration threshold is required to define the en domain of expression. Instead, en seems to be responsive to integration of Hh signaling over time, as it shares an anterior boundary with Ptc, and presumably Col, at later time points. Therefore, cells exposed to two different Hedgehog concentrations δ1 and δ2 above the switching threshold, δ, turn on Hh target gene expression at different time points but eventually activate all target genes (Territory V). (2.88 MB TIF) [file pbio.1000202.s006.tif]
