# Supplementary material for: Dynamic Interpretation of Hedgehog Signaling in the Drosophila Wing Disc
Source: PLoS Biol. 2009 Sep 29;7(9):e1000202. doi: 10.1371/journal.pbio.1000202 (PMC2744877; doi:10.1371/journal.pbio.1000202)
Supplement: Protocol S1 — Generation of intensity profiles for Figure 2E . (0.03 MB DOC) [file pbio.1000202.s007.doc]

**Protocol S1. Generating concentration profiles for data in Fig. 2E.**

The following protocol explains how the expression of Ptc was quantified to generate the concentration profiles in Fig. 2E. This process was followed with each image manually one at a time. The process was repeated independently twice in each image and the final result was averaged. For each time point, 4-7 discs were analyzed.

1. The original image was passed through a Gaussian filter (medfilt2 in Matlab).

2. Crop a 30X30 pixel box manually from the Posterior compartment of the filtered image and compute the average pixel intensity. This is the background intensity.

3. Subtract the background intensity to the whole filtered image.

4. Crop a 30X30 pixel box manually from the Anterior compartment (far from the AP boundary) of the filtered-background subtracted image and compute the average pixel intensity. This is the “Ptc basal intensity”.

5. Crop a 20X80 pixel box that crosses the AP boundary in a place that the posterior border of Ptc looks approximately straight (as the red box in the cartoon of Fig. 2E). Average the intensities of each of the 20 lines that are perpendicular to the AP boundary.

6. Divide the average intensity at each point by the “Ptc basal intensity”. This gives the intensity profiles in units of the “Ptc basal intensity”. In figure 2E, all profiles were plotted on the same relative scale.

7. Finally, the width in Fig. 2F is defined as the distance between the points that cross an intensity equal to 2 (this is twice the Ptc basal levels). Widths for the same time points were averaged along their standard deviation are plotted in Fig. 2F.
