# Supplementary material for: Dynamic Interpretation of Hedgehog Signaling in the Drosophila Wing Disc
Source: PLoS Biol. 2009 Sep 29;7(9):e1000202. doi: 10.1371/journal.pbio.1000202 (PMC2744877; doi:10.1371/journal.pbio.1000202)
Supplement: Table S1 — Parameter values used in the computer simulations. (0.09 MB DOC) [file pbio.1000202.s008.doc]

**Table S1.** Parameter values used in the computer simulations

| **Symbol** | **Description** | **Value** | **Reference** |
| --- | --- | --- | --- |
| *D* | Hh diffusion coefficient | 0.5 μm^2^s^-1^ | Kicheva et al. 2007 [1], for Wg |
| *α*_Hh_ | Hh maximal activation rate | 3.4 x 10^-3^ μMs^-1^ | See estimation |
| *α_ptc_* | *ptc* maximal activation rate | 2.7 x 10^-5^ μMs^-1^ | This study (see estimation) |
| *α_ptc0_* | *ptc* basal transcription rate | 3.8 x 10^-6^ μMs^-1^ | Casali and Struhl 2004 [2] |
| *α*_Signal_ | Signal maximal activation rate | 1.6 x 10^-4^ μMs^-1^ | Denef et al. 2000 [3] |
| *β*_Hh_ | Hh degradation rate | 3.3 x 10^-3^ s^-1^ | See estimation |
| *β*_ptc_ | *ptc* degradation rate | 1.4 x 10^-4^ s^-1^ | See estimation |
| *β*_Ptc_ | Ptc degradation rate | 1.5 x 10^-3^ s^-1^ | French and Lauffenburger 1996 [4] |
| *β*_Signal_ | ‘Signal’ degradation rate | 5.5 x 10^-4^ s^-1^ | See estimation |
| *β*Hh_Ptc | Hh_Ptc degradation rate | 1.5 x 10^-3^ s^-1^ | Torroja et al. 2004 [5] (=*β*_Ptc_) |
| *γ*Hh_Ptc | Association rate, Hh_Ptc complex | 7.15 x10^-2^ μM^-1^s^-1^ | Lander et al. 2002 [6]for Dpp |
| *T*_Ptc_ | Ptc translation rate | 3.6 x 10^-3^ s^-1^ | See estimation |
| *k_ptc_* | *ptc* half-maximal activation conc. | 0.14 μM | See estimation |
| *k*_Signal_ | [Signal] half-maximal activation | 2.135 | Casali and Struhl 2004 [2] |
| *m* | Hill coefficient (*ptc* activation) | 3 | Eldar et al. 2003 [7] |
| *n* | Hill coefficient (‘Signal’ activation) | 6.8 | Casali and Struhl 2004 [2] (see text) |

**REFERENCES:**

1. Kicheva A, Pantazis P, Bollenbach T, Kalaidzidis Y, Bittig T, et al. (2007) Kinetics of morphogen gradient formation. Science 315: 521-525.

2. Casali A, Struhl G (2004) Reading the Hedgehog morphogen gradient by measuring the ratio of bound to unbound Patched protein. Nature 431: 76-80.

3. Denef N, Neubuser D, Perez L, Cohen SM (2000) Hedgehog induces opposite changes in turnover and subcellular localization of patched and smoothened. Cell 102: 521-531.

4. French AR, Lauffenburger DA (1996) Intracellular receptor/ligand sorting based on endosomal retention components. Biotechnol Bioeng 51: 281-297.

5. Torroja C, Gorfinkiel N, Guerrero I (2004) Patched controls the Hedgehog gradient by endocytosis in a dynamin-dependent manner, but this internalization does not play a major role in signal transduction. Development 131: 2395-2408.

6. Lander AD, Nie Q, Wan FY (2002) Do morphogen gradients arise by diffusion? Dev Cell 2: 785-796.

7. Eldar A, Rosin D, Shilo BZ, Barkai N (2003) Self-enhanced ligand degradation underlies robustness of morphogen gradients. Dev Cell 5: 635-646.
