# Supplementary material for: Dynamic Interpretation of Hedgehog Signaling in the Drosophila Wing Disc
Source: PLoS Biol. 2009 Sep 29;7(9):e1000202. doi: 10.1371/journal.pbio.1000202 (PMC2744877; doi:10.1371/journal.pbio.1000202)
Supplement: Text S1 — Supporting Text and References. (0.31 MB DOC) [file pbio.1000202.s009.doc]

**SUPPORTING TEXT**

**Table of Contents:**

Justification of assumptions Page 1

Parameters Page 5

Steady-State analysis Page 8

Ratiometric model and estimation of n Page 11

Analysis of the Hh gradient in *ptc*-TPT and *ptc*+TPT discs Page 14

Supporting References Page 17

**Justification of assumptions**

We modeled Hh signaling in the wing disc using a system of nonlinear partial differential equations (Equations 1-5). The general form of the equations is similar to previous morphogen models of the wing disc [1,2,3,4,5]. Here we discuss the approximations used in the formulation of our mathematical model.

*Disc Geometry*. Wing discs in *Drosophila* are approximately flat larval tissues. We assumed that anterior-posterior (AP) patterning of the disc can be modeled as a one-dimensional system. Because Hh crosses into the anterior compartment from all positions along the AP boundary, a one-dimensional domain is a good approximation of the complicated disc geometry [6]. The subdivision of wing discs into compartments is much more than a convenient definition. The posterior compartment is defined by the expression of the selector gene *engrailed* and cell populations from different compartments do not seem to intermix during development [7]. Thus, the concepts of compartments and AP boundary are well defined in the system. The AP axis of a third instar wing discs is about 200 μm (~80 cells) long [8,9]. We assumed that the AP boundary divides this one-dimensional domain into two equal parts (of 100 μm each). From our images, this seems a fair approximation. We do not expect that any of these approximations of the disc geometry affect the conclusions of this study.

*Disc Growth*. Cells in third instar wing discs proliferate in a uniform manner, approximately one division every 8 hours [10]. Since the time-scale of cell proliferation is slow compared to morphogen gradient formation [11,12], in our model we assumed that patterning and growth can be decoupled, and discs are assumed to have a fixed size. A study suggests that the Dpp gradient in the wing disc is not affected by disc growth [13]. Since the Hh gradient specifies the source of the Dpp gradient, it is likely that the Hh gradient is also not affected by disc growth. It is unclear when the Hh gradient forms during wing disc development. However, as the range of Hh is relatively short, the results do not depend on a precise size/stage of the discs.

*Equations*. *hedgehog* is transcriptionally activated in every posterior cell by the selector gene *engrailed* and we assume that Hh is produced and secreted at a constant rate in all cells of the posterior compartment [Equation (1)]. Hh transport into the anterior compartment is a complex process and requires heparan-sulfate proteoglycans and lipoproteins [14,15], but it is mainly transported extracellularly and not by sequential rounds of dynamin-dependent endocytosis/exocytosis [16,17]. We assume that this transport process can be modeled as an effective diffusion process [Equation (1)]. This seems to be a good approximation [4,6].

Ligand-Receptor binding reaction is modeled as a mass-action law; in this case, the rate of Hh_Ptc formation is proportional to the product of [Hh] and [Ptc] [Equations (1-3)]. Because upon Hh binding to Ptc, the Hh_Ptc complex is internalized and targeted to degradation [16], we modeled the Hh+Ptc🡪 Hh_Ptc reaction as irreversible, i.e., once the Hh_Ptc complex is formed and internalized, the Ptc receptor cannot be reused and the Hh ligand cannot be re-secreted [Equation (1)]. This assumption is supported by experimental data [16], but in any case, assuming that the reaction Hh_Ptc🡪Hh+Ptc occurs at a slow rate (compared to the inverse reaction) it does not affect the conclusions of this study (data not shown). We modeled the distribution of the receptor Ptc, both mRNA and protein, in space and time. *ptc* is constitutively present at low levels in the anterior compartment, but in addition, it is transcriptionally upregulated by Hh signaling. Hh-dependent *ptc* transcription is modeled using a Hill function that depends on the levels of signaling activity, here represented by the variable [Signal] [Equation (2)]. In equation (3), the rate of *ptc* translation is assumed proportional to [*ptc*] as in other studies [18].

A molecular model of how Smo activation depends on Ptc and Hh_Ptc is missing, but a study has reported the ability of Hh-Ptc complexes to titrate the inhibition of Ptc on signal activation [19]. We use these phenomenological observations to model the rate of “Signal” activation as a Hill function of the [Hh_Ptc] to [Ptc] ratio [Equation 5]. Although the choice of this Hill function is not directly justified by chemical reactions, experimental data suggests that the rate of “Signal” activation is a saturation curve that depends on the [Hh_Ptc] to [Ptc] ratio [19] (see also Figure 1D). Numerical simulations suggest that other choices of sigmoid curves do not affect our conclusions (data not shown). We do not include equations for other Hh target genes in Figure 1B because their profiles are only outputs of the system and are not required for the analysis.

*Initial and Boundary Conditions*. The initial conditions of the system are unclear, since many of the genes involved in Hh signaling may be expressed early in wing disc development. Nevertheless, we administered the initial conditions which are demonstrated sufficient to rescue patterning to approximate wild-type conditions when the system is reinitialized artificially using the *hh* temperature sensitive mutation *hh^ts2^* [20]. Since *ptc* is expressed in a Hh-independent way at low levels in the anterior compartment, we set the following initial conditions (I.C.) for [*ptc*] and [Ptc],

**(I.C. 1)** [*ptc*](*x*,0) = = for *x* > 0, and zero otherwise,

**(I.C. 2)** [Ptc](x,0) = .

All other terms in the mathematical model are set to zero initially. Unlike other theoretical studies [3,6], we have explicitly included the posterior compartment in our model and simulations. This simplifies (and makes irrelevant) the choice of the posterior boundary condition. Also, because the range of the Hh gradient is short compared to the size of the anterior compartment, the results are largely independent of the choice of the anterior boundary condition. In our simulations, we use zero-flux boundary conditions boundary conditions (B.C.) at *x* =100 μm,

**(B.C.)**  .

**Parameters**

The parameters values used in the computer simulations of Equations (1-5) are reported in Table S1. Parameter values were either extracted from the literature, or estimated from experimental or empirical measurements. In this section, we discuss the estimation of those parameters that are not extracted explicitly from the literature. Third instar wing disc cells have an approximate diameter of 2.5 μm and an approximate volume of litres [6]. When parameters are reported in other units, we use these factors to convert parameters into units of Molar.

Hh effective diffusion coefficient *(D)*. The Hh diffusion coefficient has not been measured. For Dpp and Wingless in the wing disc, reported values are 0.1 and 0.5 μm^2^/s, respectively. Hh transport seems more similar to that of Wingless, which also requires lipoparticles [15]. Therefore, we estimated *D* = 0.5 μm^2^/s.

Hh production-secretion rate *(α*_Hh_*).* We assumed that extracellular levels of Hh are about 1μM in the posterior compartment; this is similar to the levels of Dpp in the source of the Dpp gradient. Since the maximum levels of Hh, Hh_Max_, are given by Hh_Max_, we have that *α* _Hh_= (1 μM)*β* _Hh_.

Hh-Ptc association rate *(γ *Hh_Ptc*).* We use *γ *Hh_Ptc = 0.12 μM^-1^ s^-1^ as used for analysis of the Dpp system [6].

Hh degradation rate *(β *_Hh_*)*. The characteristic space-constant, λ, of an exponential concentration gradient is formally defined as the distance at which the concentration drops by a factor of 1/e of its maximal intensity. For a morphogen gradient established by diffusion (D) and linear degradation (*β*), we have . However, if in addition, Hh is degraded by binding its receptor, we have that the space constant is where <Ptc> is the average unoccupied Ptc receptors in the Hh operating domain. Based on computer simulations, we estimated an average of 65% unoccupied Ptc. Studies have reported *λ*_Hh_ 3.5 μm [21], which permits the following estimation, s^-1^.

*ptc* translation rate (T_Ptc_). The control of mRNA translation is precisely regulated in different eukaryotic cells. For example, the rate of rabbit globin translation when globin mRNA is injected into Xenopus oocytes is 110 globin proteins per mRNA molecule per hour [22], and this number does not appear to change in different embryonic stages or cell types [23]. Since rabbit globin mRNA is 650 base pairs (bp) [24], we estimated that the rate of translation in eukaryotes is about 20 bp/sec. Thus, T_Ptc_ is obtained using this estimated rate of translation as well as information that the *ptc* mRNA is 5535 bp in length [(www.flybase.org](http://www.flybase.org/)).

Ptc and Hh_Ptc degradation rates *(β*_Ptc_ and *β*Hh_Ptc*)*. It has been proposed that Ptc internalization and degradation is independent of Hh binding [25]. Therefore, we assume that *β*_Ptc_ = *β*Hh_Ptc. We use the receptor degradation rate measured in other vertebrate receptors which is consistent with the rapid turnover of Ptc when Hh signaling is shut off in *hh^ts2^* discs [8].

*ptc* transcription rate (*α_ptc_* and *α_ptc0_*)*.* In our experiments, Ptc is detected as soon as 2 hours after Hh signal is recovered (Figure 2E) suggesting that [*ptc*] levels can build up within 2 hours. Maximum receptor levels in this system have been estimated at approximately 1,600 per cell [6], this gives 0.48 μM. Therefore, we can estimate *ptc* maximum levels as . If these levels can be reached within approximately 2 hours then the rate of *ptc* activation is . Finally, the Hh-independent levels in the anterior compartment are about 7-fold lower than in the Hh-induced *ptc* domain [19]. Therefore, we set .

*ptc* degradation rate *(β_ptc_)*. From the previous computation, .

”Signal” activation rate *(α*_Signal_*)*. We assume that signal activity depends on the rates of Smo phosphorylation. The maximum levels of pSmo are assumed to be 0.3 μM. Denef et al. [26] showed that a pSmo replaces unphosphorylated Smo within 30 minutes after exposure to Hh in cell culture [26]. Therefore, we estimate .

”Signal” degradation rate *(β*_Signal_*).* From the last expression, .

[Signal] level required for *ptc* half-maximal activation *(k_ptc_)*. We simulated the system and found a value of *k_ptc_* to support a *ptc* domain which is 5 cells wide.

Hh_Ptc:Ptc ratio required for half-maximal activation of Signal *(k*_Signal_*)*. Casali and Struhl suggested that this ratio is approximately two [19].

Hill coefficients (m and n). We set m=3 [2]. In the text (see also discussion below), we estimated n > 6.8, (n > 4.0 in the more stringent scenario). In all the simulations, however (except for Figure 1C), n was set to 6.8.

**Steady-State analysis**

At steady-state, it is possible to reduce the full model [Equations (1-5)] to a single equation of [Hh]. Setting the right-hand side of equations (1-5) to zero, and making the necessary algebraic substitutions we obtain the following boundary-value problem

with , , and , and satisfying the boundary conditions

Equation (S1) is still difficult to solve analytically but can be used to compute steady-state profiles numerically. The solution of the full model [Equations (1-5)] approaches to the steady-state solution of the last equation and are practically undistinguishable after 8 hours of simulation time (data not shown).

At steady-state, it is also possible to write each variable as an explicit function of [Hh]_ss_. An example is the steady-state profile of [Signal]_ss_ as a function of the extracellular gradient [Hh]_ss_ [Equation (6)]. Figure 1E shows that based on the estimation of the parameter *n* (Figure 1D and next section), the shape of the [Signal]_ss_ profile is step-like. In this situation, the parameter ε defined in equation (6) can be interpreted as a ‘switching threshold’ (see below for discussion of this concept). If a cell experiences an extracellular concentrations of Hh higher than ε, Hh signaling will be activated in that cell, while if [Hh]_ss_ < ε, then that cell will not be responsive to the Hh pathway. This binary behavior of the system is, of course, only strictly true when . In practice, this is only an approximation and therefore, cells that experience extracellular Hh levels similar to ε will produce ‘intermediate’ levels of “Signal”. Therefore, it is appropriate to ask what is the size of the region expressing these ‘intermediate’ levels of “Signal” at steady-state when *n* > 6.8. A simple inspection of Figure 1E suggests that [Signal]_ss_ levels change from nearly maximal to nearly minimal within < 3 cell diameters. However, it is not clear what the range of [Signal]_ss_ is that can activate Hh signalling *in vivo*. Morphogen studies in *Xenopus* suggest that 100 active receptors is about the lowest signaling concentration that that cells can respond to [27]. If we consider similar numbers for the signal transducer pSmo in our system (which is also a transmembrane molecule) this is about 0.03 μM. In our simulation results of Figure 1E, a cell at position 9 would express already lower levels of [Signal] at steady-state. Thus, according to this analysis, we estimate that [Signal] levels drop from nearly maximal to undetectable in less 3 cells. Because this estimation depends on many uncertain parameters, we cannot strictly rule out that some concentration-dependent effects contribute to some extent to the final output. In fact, the small overlap of Col and dppZ detected in Figure 3F may be due to a slight concentration-dependent difference between the two target genes.

The steady-state interpretation of the Hh extracellular gradient in terms of equation (6) requires some additional discussion. Since [Hh] refers to free unbound Hh concentration, strictly speaking, equation (6) is not the relationship that describes how cells ‘translate’ extracellular levels into signaling outputs because cells probably do not have another way of sensing extracellular Hh concentrations other than by using the receptor Ptc. This does not affect any of the predictions of the model. However, it is important to clarify the meaning of some parameters. For example, the interpretation of ε as the ‘switching threshold’ is somehow artificial, because cells (presumably) do not have a way to ‘measure’ unbound extracellular [Hh]. A more appropriate definition of ‘switching threshold’ may be obtained using an extracellular gradient that cells can sense. Since, all steady-state variables can be written as a function of [Hh]_ss_, we can define more realistic ‘switching thresholds’. For example, we can write [Ptc]_ss_ as a function of [Hh]_ss_ and set the switching threshold to be [Ptc]_ss_(ε). The switching threshold can also be defined in terms of [Hh_Ptc]_ss_, , or other combinations. Using the ratiometric model, the natural choice of switching threshold is given by

. (S2)

Note that δ and ε are linearly proportional. Indeed, the last equation is just a particular case of a more general property of the system, namely, at steady-state, equation (4) can be written in the form

= [Hh]_ss_. (S3)

Therefore, defining the switching threshold as ε or δ is equivalent. Nonetheless, δ (and not ε) is used as ‘switching threshold’ in Figures 3 and 5. In any case, we insist that other choices do not affect the concept or the conclusions of this study.

**Ratiometric model and estimation of n**

Our mathematical analysis depends on the ratiometric model of Hh signaling interpretation [19]. Since this model has caused some controversy in the literature [28], it is appropriate to discuss possible problems that may arise in this treatment. The ratiometric interpretation of Hh signaling is a phenomenological model, this is, it is based on observations about target gene expression, but does not provide a molecular mechanism of Hh signal activation. In the past, it was thought that the levels of free (unliganded) Ptc would control the activity of the signal. In this scenario, signaling activity would not change as long as levels of free Ptc are maintained constant. The ratiometric model is introduced to explain the effect of [Hh_Ptc] levels in cells with a constant background of free Ptc [19]. To a first approximation, the ratiometric model explains how cells would correct the interpretation of a certain concentration of [Ptc] levels when also expressing [Hh_Ptc], but experiments in vertebrates or in different conditions suggest that the ratiometric model is probably not applicable in general. For example, the ratiometric model may be sensitive to total levels of Ptc, as experiments in vertebrates do not seem to obey the predictions of the model [29]. If so, the ratiometric model may not explain some mutants such as *ptc* overexpression. Despite these difficulties, the ratiometric model explains Hh patterning to a good approximation under normal physiological conditions. Because our analysis is based on the wild-type and many important details about Hh transduction are unknown, we argue that for the purpose of this study, the ratiometric model may be used as a phenomenological model of Hh signal interpretation.

An additional consideration is the use of the measurements from Casali and Struhl [19] to estimate the value of n. Here, we give a formal mathematical argument of the lower-bound estimation of n in Figure 1D. The basic idea is that n has a geometric meaning in the graph of the Hill function for which n is the Hill coefficient; it is a measure of the steepness of the Hill function. The following proposition shows that, given two points in the curve of the Hill function, it is possible to estimate a lower-bound estimate of n.

*Proposition: Consider the function* . *Let x, y be real numbers and without loss of generality take xy. Then,*

*(If x=y, the left hand side is understood as the limit, i.e. ).*

*Proof. By the intermediate value theorem of calculus there is xzy such that*

*A simple computation shows that the minimum of F’(x) is reached when x=k. This completes the proof.*

Let . The experiments of Casali and Struhl [19] provide two data points in the graph of F as they made clones of *ptc*^-^ cells in the anterior compartment expressing different amounts of the [Hh_Ptc]/[Ptc] ratio. For , they found no detectable activation of Hh target genes, and for , they found high expression of Hh outputs. We can therefore, apply the proposition above to obtain a lower-bound estimate of n. We assumed that F(1.6)=5% and F(2.7)=95% to represent no and full activation of the pathway up to a 5% detection error (95%). Unfortunately, Casali and Struhl did not compare gene expression levels in the clone to wild-type gene expression in the same disc, but both Col and a reporter of *dpp* appear strongly expressed in these clones [19]. Also, it is not clear whether or not signaling activity is maximal for , since Casali and Struhl did not report whether or not *engrailed* was expressed in clones expressing this ratio. Therefore, the accuracy of the estimate F(2.7) = 95% is unknown. However, reasonable deviations of this choice do not affect our conclusions. For example, setting F(2.7) = 80% gives the lower-bound estimate of n to be 5.7. As other caveats can be found in the use of these data for the estimation of the value of n, we insist that to give an accurate and reliable value of n is not a result or goal of this study. Instead, our estimation of n suggests that the hypothesis that the steady-state [Signal] profile has a step-like shape is reasonable and consistent with experimental findings.

**Analysis of the Hh gradient in *ptc*-TPT and *ptc*+TPT discs**

Hh signaling in *ptc*-TPT discs can be simulated using our mathematical model, with the exception of equation (2), which is replaced by

, (S4)

and the initial condition (I.C.1) is replaced by [*ptc*](*x*,0) = . Compared to equation (2), the last equation eliminates all wild-type *ptc* expression and models ubiquitous expression of *ptc* as introduced by the TPT transgene. Chen and Struhl estimated that two copies of the TPT transgene introduce Ptc levels that are less than twofold compared to the maximal levels of Ptc near the AP boundary [30]. Therefore, we set in the numerical simulations of Hh signaling in *ptc*-TPT discs.

Regarding the levels introduced by the TPT transgene, it is important to clarify that in a previous report it was observed that dppZ expands further anteriorly with respect to the wild-type pattern (reported as data not shown in ref. [30]). However, in our own experiments we observe that the dppZ domain is slightly reduced with respect to wild-type (Figure 3B,E). This apparent discrepancy may be due by one of the following reasons. First, it is possible that the Chen and Struhl [30] used another insertion of the TPT transgene, for example, one on the second chromosome. Or second, the TPT transgene may have acquired mutations in the past decade that affected the performance of the original transgene. In any case, the predictions of the Overshoot Model are not affected by the strength of the transgene. The Overshoot Model predicts the Col and dppZ patterns to overlap regardless of whether each pattern expands or reduces with respect to the wild-type.

Although the profiles shown in Figure 3G,H are intended as illustrations of the predictions of the Classical Morphogen Model vs. Overshoot Model in different genetic backgrounds, it is illustrative to estimate the expected size of the dppZ domain that would not overlap with the Col pattern in *ptc*-TPT discs according to the interpretation of the Classical Morphogen Model. One way of doing this is to measure the size of the Col vs. dppZ domain in wild type discs and use the numerical simulations to predict the expected non-overlapping region in *ptc*-TPT discs. However, this estimate will depend in the parameter values used in the simulations, as well as in our accuracy to measure the dppZ domain, which is difficult to estimate reliably because unlike Col, in wild-type discs the anterior boundary of the dppZ is not sharp (Figure 3C).

Another way of estimating the difference in Col and dppZ patterns according to the Classical Morphogen model interpretation is to compare the shape of the Hh gradient in the region anterior to the Ptc/Col domain (*x* > *x*_ptc/col_, with *x*_ptc/col_ denoting the position of the anterior border of Ptc/Col, which we assume is the same throughout this analysis). As Ptc is not upregulated by Hh signaling in this region, we can assume that Ptc levels are approximately constant (denoted by [Ptc]_A_) so that equation (1) at steady-state is linear and given by

, (S5)

subject to the boundary condition [Hh]_SS_(*x*_ptc_)=T_1_, which assumes that the Ptc boundary is set by a fixed concentration threshold, denoted by T_1_. Equation (S5) has the following solution,

(*x* > *x*_ptc/col_), (S6)

where is the characteristic length of the gradient. In the context of the Classical Morphogen Model, λ is actually a good representation of the width of the non-overlapping domain. Equations (S5-S6) are also valid in *ptc+*TPT and *ptc*-TPT discs provided that the constant [Ptc]_A_ is adjusted appropriately. Thus, we can compare what are the expected differences in the Col and dppZ domains in these backgrounds according to the Classical Morphogen Model. If we denote [Ptc]_0_ the anterior Ptc levels in wild-type discs, and assume that the levels of Ptc introduced by the TPT transgene can be denoted by [Ptc]_TPT_ (from measurements of an early study using the TPT transgene, it can been estimated that [Ptc]_TPT_/[Ptc]_0_=1.4), then the predicted differences in Col/Ptc and dppZ domains in different mutant backgrounds are given by:

(S7)

Thus, equations (S7) provide a quantitative proof of our predictions in Figure 4A-C, namely, λ*_ptc-_*_TPT_ > λ*_ptc_*_+TPT_ independently on the Ptc levels expressed by the TPT transgene. Our data in Figure 4F’ show that experimentally λ*_ptc+_*_TPT_ is at least 3 cells wide, suggesting that the non-overlapping region in *ptc*-TPT would be larger than 3 cells. This prediction is clearly not consistent with the observations in Figure 3F’, demonstrating that the patterns in these mutant backgrounds cannot be explained by the Classical Morphogen Model. Thus, this analysis provides strong support for the Overshoot Model over the Classical Morphogen Model in this system.

**SUPPORTING REFERENCES**

1. Lander AD (2007) Morpheus unbound: reimagining the morphogen gradient. Cell 128: 245-256.

2. Eldar A, Rosin D, Shilo BZ, Barkai N (2003) Self-enhanced ligand degradation underlies robustness of morphogen gradients. Dev Cell 5: 635-646.

3. Bollenbach T, Kruse K, Pantazis P, Gonzalez-Gaitan M, Julicher F (2005) Robust formation of morphogen gradients. Phys Rev Lett 94: 018103.

4. Gunbin KV, Omelyanchuk LV, Kogai VV, Fadeev SI, Kolchanov NA (2007) Model of the reception of hedgehog morphogen concentration gradient: comparison with an extended range of experimental data. J Bioinform Comput Biol 5: 491-506.

5. Nahmad M, Glass L, Abouheif E (2008) The dynamics of developmental system drift in the gene network underlying wing polyphenism in ants: a mathematical model. Evol Dev 10: 360-374.

6. Lander AD, Nie Q, Wan FY (2002) Do morphogen gradients arise by diffusion? Dev Cell 2: 785-796.

7. Garcia-Bellido A, Merriam JR (1971) Genetic Analysis of Cell Heredity in Imaginal Discs of Drosophila melanogaster. Proc Natl Acad Sci U S A 68: 2222-2226.

8. Held LI (2002) Imaginal discs : the genetic and cellular logic of pattern formation. Cambridge [England] ; New York: Cambridge University Press. xv, 460 p. p.

9. Bollenbach T, Pantazis P, Kicheva A, Bokel C, Gonzalez-Gaitan M, et al. (2008) Precision of the Dpp gradient. Development 135: 1137-1146.

10. Gonzalez-Gaitan M, Capdevila MP, Garcia-Bellido A (1994) Cell proliferation patterns in the wing imaginal disc of Drosophila. Mech Dev 46: 183-200.

11. Teleman AA, Cohen SM (2000) Dpp gradient formation in the Drosophila wing imaginal disc. Cell 103: 971-980.

12. Kicheva A, Pantazis P, Bollenbach T, Kalaidzidis Y, Bittig T, et al. (2007) Kinetics of morphogen gradient formation. Science 315: 521-525.

13. Hufnagel L, Teleman AA, Rouault H, Cohen SM, Shraiman BI (2007) On the mechanism of wing size determination in fly development. Proc Natl Acad Sci U S A 104: 3835-3840.

14. Han C, Belenkaya TY, Wang B, Lin X (2004) Drosophila glypicans control the cell-to-cell movement of Hedgehog by a dynamin-independent process. Development 131: 601-611.

15. Panakova D, Sprong H, Marois E, Thiele C, Eaton S (2005) Lipoprotein particles are required for Hedgehog and Wingless signalling. Nature 435: 58-65.

16. Torroja C, Gorfinkiel N, Guerrero I (2004) Patched controls the Hedgehog gradient by endocytosis in a dynamin-dependent manner, but this internalization does not play a major role in signal transduction. Development 131: 2395-2408.

17. Su VF, Jones KA, Brodsky M, The I (2007) Quantitative analysis of Hedgehog gradient formation using an inducible expression system. BMC Dev Biol 7: 43.

18. von Dassow G, Meir E, Munro EM, Odell GM (2000) The segment polarity network is a robust developmental module. Nature 406: 188-192.

19. Casali A, Struhl G (2004) Reading the Hedgehog morphogen gradient by measuring the ratio of bound to unbound Patched protein. Nature 431: 76-80.

20. Strigini M, Cohen SM (1997) A Hedgehog activity gradient contributes to AP axial patterning of the Drosophila wing. Development 124: 4697-4705.

21. Kicheva A, Gonzalez-Gaitan M (2008) The Decapentaplegic morphogen gradient: a precise definition. Curr Opin Cell Biol 20: 137-143.

22. Gurdon JB (1974) The control of gene expression in animal development. Cambidge, Mass.: Harvard University Press.

23. Lodish HF (1976) Translational control of protein synthesis. Annu Rev Biochem 45: 39-72.

24. Gaskill P, Kabat D (1971) Unexpectedly large size of globin messenger ribonucleic acid. Proc Natl Acad Sci U S A 68: 72-75.

25. Gallet A, Therond PP (2005) Temporal modulation of the Hedgehog morphogen gradient by a patched-dependent targeting to lysosomal compartment. Dev Biol 277: 51-62.

26. Denef N, Neubuser D, Perez L, Cohen SM (2000) Hedgehog induces opposite changes in turnover and subcellular localization of patched and smoothened. Cell 102: 521-531.

27. Dyson S, Gurdon JB (1998) The interpretation of position in a morphogen gradient as revealed by occupancy of activin receptors. Cell 93: 557-568.

28. Zhu AJ, Scott MP (2004) Incredible journey: how do developmental signals travel through tissue? Genes Dev 18: 2985-2997.

29. Taipale J, Cooper MK, Maiti T, Beachy PA (2002) Patched acts catalytically to suppress the activity of Smoothened. Nature 418: 892-897.

30. Chen Y, Struhl G (1996) Dual roles for patched in sequestering and transducing Hedgehog. Cell 87: 553-563.
